# Targeting PSMD14 increases chemotherapeutic sensitivity to gemcitabine by inhibiting the WNT/β-catenin pathway and glycolysis in pancreatic cancer

**DOI:** 10.1038/s41419-026-08958-w

**Published:** 2026-08-03

**Authors:** Kun Cai, Shiyu Chen, Guoqing Ma, Wenhao Wu, Songsheng Wang, Baoku Quan, Canhui Jin, Yongqiang Wang, Zhiwei He, Tianbao Wang

**Affiliations:** 1 Department of Gastrointestinal Surgery, South China Hospital, Medical School, Shenzhen University, Shenzhen, P. R. China; 2 Guangdong Key Laboratory for Biomedical Measurements and Ultrasound Imaging, National-Regional Key Technology Engineering Laboratory for Medical Ultrasound, School of Biomedical Engineering, Shenzhen University Medical School, Shenzhen, P. R. China; 3 South China Hospital, Medical School, Shenzhen University, Shenzhen, P. R. China; 4 Department of Hepatobiliary Surgery, Shenzhen University General Hospital & Shenzhen University Clinical Medical Academy Center, Shenzhen University, Shenzhen, P. R. China; 5 Department of Hepatobiliary Surgery, The Affiliated Hospital of Guizhou Medical University, Guizhou Medical University, Guiyang, P. R. China

**Keywords:** Pancreatic cancer, Prognostic markers

## Abstract

Chemotherapy resistance is one of the important reasons for the low 5-year survival rate of patients with pancreatic cancer. Elucidating the mechanism of chemotherapy resistance is an important breakthrough direction for improving the prognosis. Here, PSMD14, a deubiquitinating enzyme frequently overexpressed in human pancreatic cancer, was demonstrated to mediate chemoresistance principally by activating the WNT/β-catenin pathway. Mechanistically, PSMD14 activated the WNT/β-catenin pathway by removing the K63-linked ubiquitination of DVL1 and promoting its translocation from the cytoplasm to the membrane, consequently facilitating the aerobic glycolysis and contributing to the chemoresistance of pancreatic cancer cells. Furthermore, activation of the WNT/β-catenin pathway promoted the transcription of PSMD14, and the PSMD14 inhibitor capzimin improved the sensitivity of pancreatic cancer cells to gemcitabine in cells, organoids, and mouse models. In conclusion, PSMD14 plays a critical role in regulating the activity of WNT/β-catenin and the sensitivity of pancreatic cancer to gemcitabine, suggesting that PSMD14 appears to be a promising therapeutic target for pancreatic cancer.

## Introduction

Pancreatic cancer (PC) is a malignant tumor of the digestive system with a 5-year survival rate of only 13% [1]. Owing to latent and nonspecific clinical symptoms, most patients are already in the tumor progression stage at the time of initial diagnosis [2]. As surgical treatment alone offers limited efficacy, chemotherapy is an important method for the treatment of PC [3, 4]. Gemcitabine has long been one of the first-line standard chemotherapy regimens for patients with advanced or metastatic pancreatic ductal adenocarcinoma [5]. However, gemcitabine resistance is caused by multiple molecular mechanisms, making the effects of chemotherapy far from satisfactory [6]. Further study of the molecular mechanism of gemcitabine resistance and identification of ways to improve the therapeutic effect of gemcitabine are highly important for improving the overall prognosis of PC.

The WNT/β-catenin signaling pathway, also known as the canonical WNT/β-catenin pathway, functions primarily by regulating the transcription of target genes [7, 8]. Numerous studies have confirmed that abnormal activation of WNT/β-catenin-mediated transcription is closely associated with the malignancy, metabolic reprogramming, and treatment resistance of PC [9–12]. An association between its abnormal activation and gemcitabine resistance has also been reported [13–15]. Further study of the mechanism of action of this pathway in PC resistance to gemcitabine therapy will help in the development of strategies to improve the efficacy of drug therapy.

Ubiquitination, in which the C-terminal glycine of ubiquitin is covalently linked to specific lysine residues on a target protein, is involved in processes such as protein degradation, epigenetic regulation, DNA repair, protein interaction, endocytosis, and protein trafficking [16]. It is one of the most common posttranslational modifications (PTMs) in cells. Like other PTMs, ubiquitination is a tightly regulated, reversible process that can be reversed by a large group of proteases, called deubiquitinating enzymes (DUBs), which regulate the ubiquitination level of the target protein precisely. DUBs not only inhibit the ubiquitination process but also promote the ubiquitination process by decomposing ubiquitination inhibitors, recycling ubiquitination molecules, and proofreading the ubiquitination process, thus forming a complex regulatory network together with the ubiquitination system.

In this study, we identified PSMD14, a 19S proteasome-associated deubiquitinating enzyme, as a key regulator of WNT/β-catenin signaling pathway activity in PC. Furthermore, we found that PSMD14 activated the WNT/β-catenin signaling pathway to promote the transcription of glycolysis-related target genes by removing the ubiquitination of DVL1, thereby reducing the killing effect of gemcitabine on PC cells. Moreover, the transcription of PSMD14 is also regulated by the activity of the WNT/β-catenin signaling pathway, which forms a positive feedback loop. More importantly, genetic and pharmacologic inhibition of PSMD14 markedly enhanced the therapeutic effect of gemcitabine on PC cells and organoids and effectively prolonged the overall survival of PC model mice. This study revealed that PSMD14 influences the chemotherapeutic efficiency of gemcitabine by regulating the WNT/β-catenin signaling pathway and provides a promising therapeutic strategy to alleviate resistance to gemcitabine chemotherapy in PC.

## Materials and methods

### Cell culture

The hTERT-HPNE, AsPC-1, BxPC-3, MIA PaCa-2, PANC-1, PSN-1, and SW1990 cell lines were purchased from the American Type Culture Collection (ATCC) and were cultured in RPMI 1640 medium or DMEM supplemented with 10% fetal bovine serum (FBS). The cells were cultured at 37 °C in an incubator with 5% CO_2_. All the cell lines had been authenticated through STR profiling and confirmed to be mycoplasma-free.

### TOP/FOP Flash

A total of 5 × 10^3^ cells were seeded in 96-well plates, incubated for 12 h, and then transfected with 50 ng of TOP flash reporter plasmid (Beyotime, #D2501) and FOP flash reporter plasmid (Beyotime, #D2503). Forty-eight hours after transfection, a Firefly Luciferase Reporter Gene Assay Kit (Beyotime, #RG006) was used to detect luciferase activity, and the TOP/FOP ratio was calculated to determine WNT/β-catenin pathway activity.

### Western blot

The cells were lysed using RIPA buffer (BOSTER, #AR0105) supplemented with protease and phosphatase inhibitors. Then, the cell lysis solutions were centrifuged, the upper clear liquid was transferred, and the protein concentrations were determined with a BCA Kit (BOSTER, #AR0197). When the membrane or nuclear proteins needed to be separated, a Membrane and Cytosol Protein Extraction Kit (Beyotime, #P0033) or Nuclear and Cytoplasmic Protein Extraction Kit (Beyotime, # P0027) was used. The proteins were subsequently denatured, separated via SDS‒PAGE, electrotransferred to a PVDF membrane, incubated with primary antibody, incubated with secondary antibody, and visualized via enhanced chemiluminescence (ECL) reagent (Biosharp, #BL520A) with a chemiluminescence imaging system (Tanon-5200). The antibodies used are listed in Table S1 and the full-length uncropped original western blots were uploaded as “Supplemental Material-Full-length western blots”.

### Immunofluorescence (IF)

The slides were fixed with 4% paraformaldehyde (PFA) or −20 °C methanol, permeabilized with 0.3% Triton, blocked with 5% BSA, and incubated with primary antibodies at 4 °C overnight. After incubation with a fluorescence-labeled secondary antibody, the cell nuclei were counterstained with DAPI. Images were subsequently collected and analyzed.

### Cell transfection and lentiviral infection

PSMD14 lentivirus, sh-PSMD14 lentivirus, sh-*Psmd14* lentivirus, and their corresponding control lentiviruses were obtained from Genechem. To construct stable-expressing cell lines, the cells were cultured with puromycin (1 μM, Invitrogen) for 2 weeks after lentiviral infection for 48 h. Small interfering RNAs (siRNAs) targeting CTNNB1 were obtained from RiboBio. The target sequences of the relevant genes used are listed in Table S2. The plasmids used in the study were obtained from Genechem. Transient transfection of plasmids or siRNAs was performed using Lipo3000 (Invitrogen) following the manufacturer’s guidelines.

### RNA extraction and RT‒qPCR

Total RNA was isolated from the cells and tissues using the TRIzol reagent (Invitrogen, #10296010) and quantified via a NanoDrop ND1000. The RNA was reverse-transcribed into cDNA through the PrimeScript™ RT reagent Kit (Takara, #RR047). Amplification was performed on a CFX96 Touch Real-Time Fluorescence Quantitative PCR Instrument (Bio-Rad Laboratories) using TaKaRa TB Green™ Premix Ex Taq™ II (Takara, #RR420). The 2^−ΔΔCt^ method was used to quantify targeted mRNA expression, and β-actin was used as an internal control. The primers used are listed in Table S3.

### Multiplex immunohistochemistry (mIHC)

After the tissue sections were treated with xylene and graded alcohols, heat-induced antigen retrieval was performed with EDTA antigen repair solution. Then, the tissue sections were incubated with 3% hydrogen peroxide, blocking reagents, primary antibodies, and secondary antibodies for the appropriate times. After the signal of the target protein was amplified via the tyramide signal amplification (Absin, #abs50012) method, a new round of staining was performed according to the experimental plan. After all the target proteins were labeled, DAPI was used for nuclear staining, and an anti-fluorescence quenching tablet was used to seal the tissue sections. The antibodies used are listed in Table S1.

### Immunohistochemistry (IHC)

The tissue samples were fixed, embedded, sectioned, and deparaffinized. Then, the sections were treated with 3% H_2_O_2_ and blocked with 5% BSA, after which they were incubated with the corresponding antibody overnight at 4 °C. After incubation with the secondary antibody, the sections were stained with hematoxylin and diaminobenzidine. The H score was calculated as the sum of the score for staining intensity multiplied by the score for the percentage of positively stained cells, H score = ∑(pi×i), where pi represents the percentage of positive cells of the corresponding grade and i indicates the classification of positive cells (absent = 0, weak = 1, moderate = 2, and strong positive = 3). The antibodies used are listed in Table S1.

### Extracellular acidification rate (ECAR)

A total of 2 × 10^4^ cells were seeded in each well of an XF96 cell culture microplate (Seahorse, #101085). After genetic or pharmacological treatment for 48 h, components of the Glycolysis Stress Test Kit (Seahorse, #103020) were successively added to the wells of the microplates. After the data were collected with a Seahorse XFe96 Analyzer, the data were processed using Wave Desktop.

### Glucose uptake assay

A total of 1 × 10^6^ cells were seeded in a well of a six-well plate and cultured with complete culture medium. After the cells adhered to the wall, cells were cultured in low-glucose medium for 12 h. Then the Glucose Uptake Assay Kit with DTNB (Beyotime, #S0556M) was employed to detect the cells’ glucose uptake ability according to the manufacturer’s instructions.

### L-lactate production assay

A total of 1 × 10^6^ cells were seeded in a well of a six-well plate and cultured with complete culture medium. After the cells adhered to the wall, cells were cultured in serum-free, high-glucose medium for 12 h. Then the L-Lactate Assay Kit with WST-8 (Beyotime, #S0208S) was employed to detect the cells’ lactate production ability according to the manufacturer’s instructions.

### Proteasome activity assay

A total of 2 × 10^6^ cells were seeded in a 6-cm dish and cultured with complete culture medium. After the cells adhere to the wall, cells receive corresponding treatments according to different groups for 12 h. Then the Proteasome Activity Assay Kit (Abcam, # ab107921) was employed to detect the proteasome activity according to the manufacturer’s instructions.

### Clinical specimens

Pancreatic adenocarcinoma tissues and adjacent nontumor tissues were collected from patients with PC who underwent surgical treatment. These patients had not undergone any other treatment methods before surgery. The collection and use of clinical specimens complied with the Declaration of Helsinki and were approved by the Medical Ethics Committee of South China Hospital of Shenzhen University and the ethics committee of the Affiliated Hospital of Guizhou Medical University.

### Patient-derived organoids (PDOs)

Fresh pancreatic tumor tissues from surgical resections were collected, minced into small pieces, and enzymatically dissociated to release single cells. The dissociated cells were subsequently filtered through a 100-μm filter, resuspended in Matrigel, and cultured in PC organoid culture medium (Mogengel, #MG2101).

### Cell viability of PDOs

PDOs containing 5 × 10^3^ cells were seeded in each well of a 96-well plate (Absin, #abs7242). After genetic or pharmacological treatment for 48 h, an ATP Viability Assay Kit (Absin, # abs50059) was used to detect the cell activity of the PDOs according to the instructions.

### Calcein-AM/PI

PDOs containing 5 × 10^3^ cells were seeded in each well of a 96-well plate (Corning, #abs3599). After genetic or pharmacological treatment for 48 h, a Calcein-AM/PI Double Stain Kit (Absin, # abs50056) was used to detect the cell activity of the PDOs according to the instructions. Images were collected and analyzed using a high-content imaging system (PerkinElmer, Opera Phenix Plus).

### Chromatin immunoprecipitation (ChIP)

A SimpleChIP® Plus Sonication Chromatin IP Kit (Cell Signaling Technology, #56383) was used for the ChIP assay. A total of 1 × 10^7^ PANC-1 cells were fixed with 1% formaldehyde, followed by 0.1 M glycine to stop the reaction. The chromatin was then sheared into fragments of 200–1000 bp in length through ultrasonication, and the fragments were precipitated by using the corresponding primary antibody or anti-IgG antibody. The antibodies used are listed in Table S1. Finally, the coprecipitated DNA was extracted with phenol/chloroform for amplification via qPCR.

### Dual-luciferase reporter assay

The promoter regions with wild-type (WT) or mutant (MT) binding sites were inserted into the pGL4.20 luciferase reporter vector (Promega, #E675A). The cells were plated in 96-well culture plates at a density of 5 × 10^3^ cells/well for 24 h and transfected with 100 ng of DNA and 150 ng of luciferase reporter plasmid coupled with 30 ng of pGL-4.74 Renilla control plasmid (Promega, #E692A) as an internal control. The firefly luciferase activity was subsequently normalized to the Renilla luciferase activity after transfection for 48 h.

### Animal experiments

C57BL/6 mice (8 weeks old) were purchased from Huafukang Biotechnology and randomly assigned to each group (*n* = 6). Each mouse pancreas was injected with 1 × 10^6^ Panc02-luc cells. In the different groups, 100 mg/kg gemcitabine (MedChemExpress, #HY-17026) and/or 10 mg/kg capzimin (MedChemExpress, HY-110404) were intraperitoneally injected for 6 weeks. On Day 52 of the experiment, the mice were anesthetized and intraperitoneally injected with potassium salt D-luciferin (PerkinElmer, #122799). Then, image acquisition and analysis were performed.

KPC mice (14 weeks old) were purchased from the Model Organisms Center (Shanghai, China). When survival analysis was performed, 10 KPC mice were randomly assigned to each group, and after 8 weeks of drug treatment, the mice were fed normally until they died. In another set of experiments, each group contained 6 mice. On the third day after 8 weeks of drug treatment, the mice were euthanized, and pancreatic tumor tissue and organs from the mice were dissected for follow-up experiments.

### Statistical analyses

The data were analyzed using SPSS 23.0 and are presented as the means ± standard deviations (SDs). Two-tailed unpaired Student’s *t* tests were used to analyze the differences between two groups. Survival was analyzed using the log-rank test. Pearson’s coefficient was used to assess correlations. Results with *P* < 0.05 were considered statistically significant and are reported as follows: *P < 0.05; **P < 0.01; ns, not significant.

### Ethics committee approval and patient consent

The animal experiment has been approved by the Animal Experimental Ethical Inspection Form of Guizhou Medical University (No.: 2403011). All methods were performed in accordance with the ARRIVE guidelines and the Chinese Guidelines for the Care and Use of Laboratory Animals. Collection of human tissue specimens and clinical information was approved by the Ethics Committee of South China Hospital of Shenzhen University and the Ethics Committee of the Affiliated Hospital of Guizhou Medical University (No.: HNLS20260318001-A). All procedures involving human participants complied with the principles of the Helsinki Declaration. Written informed consent was obtained from all individual participants included in the study.

## Results

### siRNA library screening identified PSMD14 as a key DUB that regulates the activity of the WNT/β-catenin pathway in PC

**Fig. 1:**
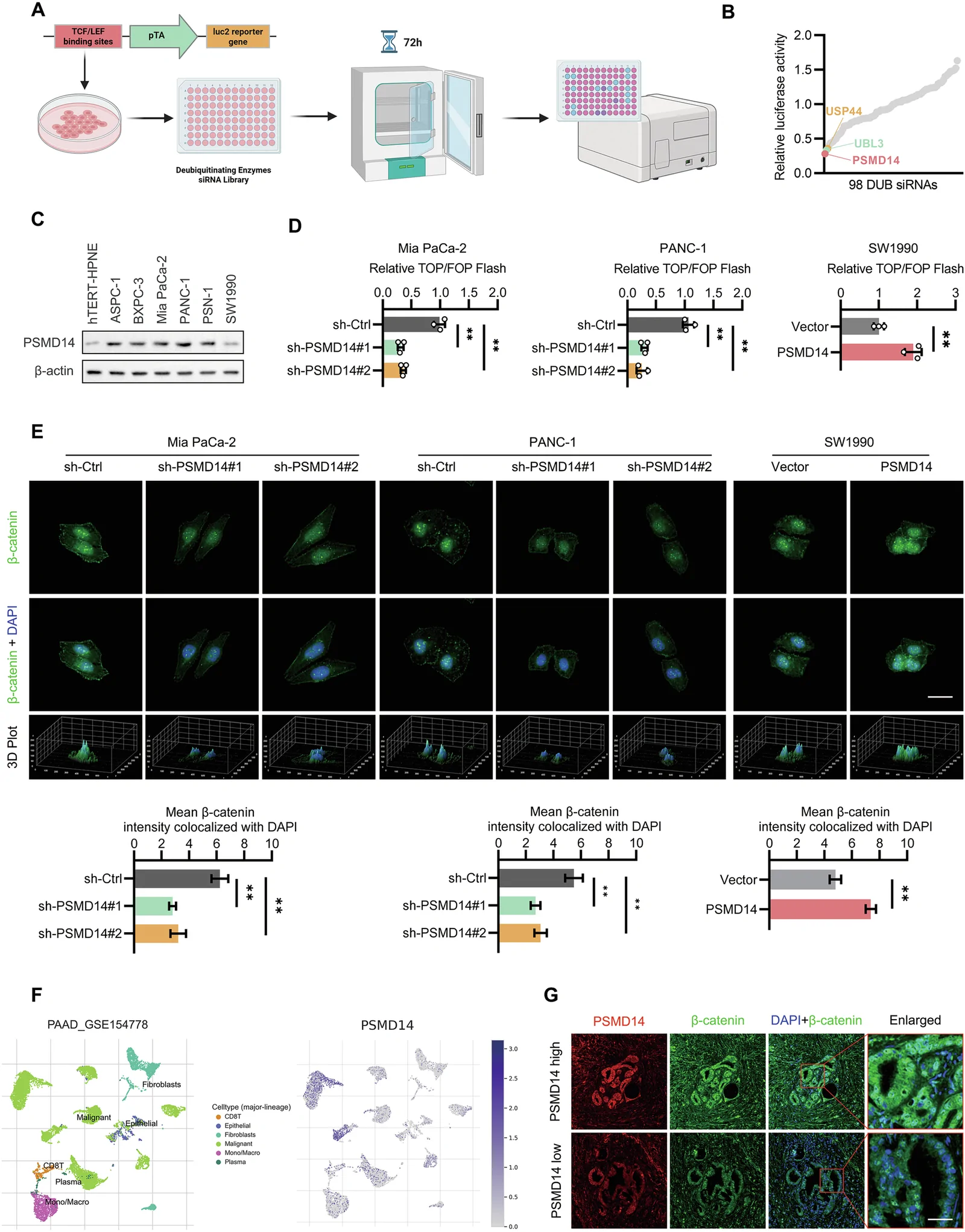
siRNA library screening identified PSMD14 as a key DUB that regulates the activity of the WNT/β-catenin pathway in pancreatic cancer. **A** Schematic diagram of DUB screening. **B** Changes in WNT/β-catenin pathway transcriptional activity after PANC-1 cells were transfected with the DUB siRNA library. **C** The protein expression levels of PSMD14 in cell lines were detected by western blotting. **D** The influence of altering PSMD14 on the WNT/β-catenin pathway was detected by a TOP/FOP assay in multiple cell lines (*n* = 3). **E** The distribution and abundance of β-catenin in cells were detected using IF. scale bar = 20 μm, (*n* = 6). **F** The abundance of PSMD14 expression in various cell subpopulations was analyzed using TISCH. **G** PSMD14 and β-catenin were detected in clinical specimens via mIHC; scale bar = 50 μm. *P < 0.05, **P < 0.01.

Abnormal activation of the WNT/β-catenin signaling pathway plays a critical role in malignant progression and treatment resistance in PC. To screen key deubiquitinating enzymes that regulate the WNT/β-catenin signaling pathway in PC, the TOP Flash or FOP Flash plasmids with a deubiquitinating enzyme library were transfected into PANC-1 cells (Fig. 1A). Finally, the TOP/FOP Flash results indicated that silencing PSMD14 inhibited TCF/LEF transcriptional activity most efficiently (Fig. 1B). After detecting the protein expression level of PSMD14 in hTERT-HPNE cells and 6 PC cell lines (Fig. 1C), we constructed PSMD14-knockdown cells (sh-PSMD14) with Mia PaCa-2 and PANC-1 cells and PSMD14-overexpressing cells (PSMD14) with SW1990 cells (Fig. S1). TOP/FOP Flash assays confirmed that PSMD14 significantly promoted the transcriptional activity of TCF/LEF in PC cells (Fig. 1D). Moreover, immunofluorescence revealed that PSMD14 increased the expression of β-catenin in the nucleus of PC cells (Fig. 1E). Analysis of the results of scRNA-seq in the TISCH2 database and clinicopathological tissue mIHC indicated that PSMD14 was localized mainly to tumor parenchymal cells and was positively correlated with the expression of β-catenin in the nucleus of tumor cells (Fig. 1F, G).

### PSMD14 is highly expressed in PC patients and is associated with poor prognosis

**Fig. 2:**
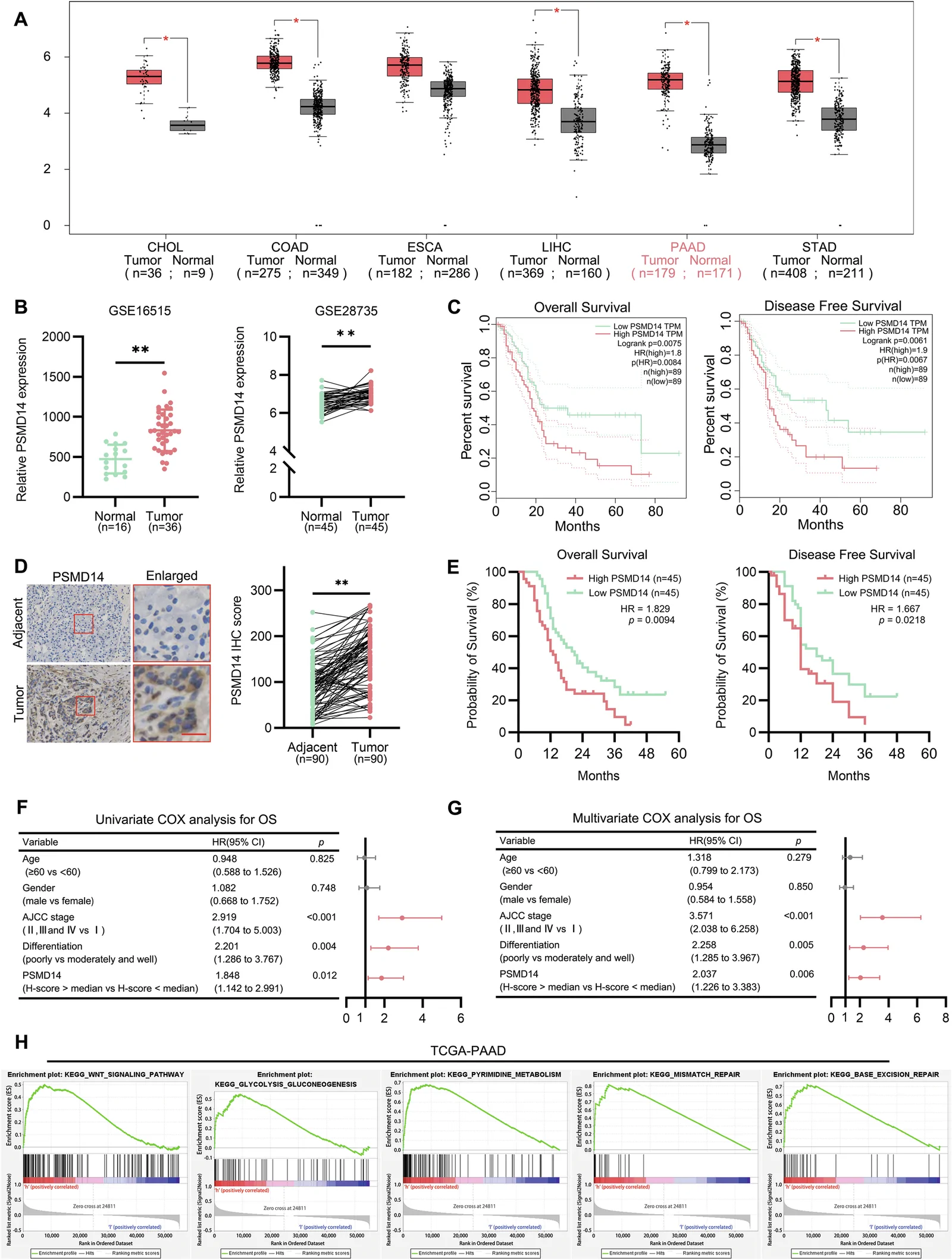
PSMD14 is highly expressed in pancreatic cancer patients and is associated with poor prognosis. **A** The transcriptional levels of PSMD14 in various digestive system tumor tissues and nontumor tissues were analyzed in the TCGA and GTEx databases. **B** The transcriptional levels of PSMD14 were analyzed in pancreatic tumor tissues and nontumor tissues on the basis of the datasets GSE16515 and GSE28735. **C** The relationships between the transcriptional level of PSMD14 in the tumor tissues of pancreatic cancer patients and the overall survival and disease-free progression survival of patients were analyzed via the TCGA database. **D** The protein expression of PSMD14 in pancreatic cancer tumor tissues and nontumor tissues was detected via IHC in clinical samples; scale bar = 50 μm (left panel). H-scores of 90 clinical specimens (right panel). **E** Analysis of overall survival and disease-free survival of pancreatic cancer patients with different H-scores of PSMD14. **F** Univariate Cox analysis for OS in 90 patients with pancreatic cancer. **G** Multivariate Cox analysis for OS in 90 patients with pancreatic cancer. **H** GSEA of pancreatic cancer data from the TCGA-PAAD dataset. *P < 0.05, **P < 0.01.

Comprehensive analysis of GTEx and TCGA data revealed that the transcription of PSMD14 in tumor tissues was integrally greater than that in normal tissues in a variety of digestive system tumors, including PC (Fig. 2A). The results of the analysis of the GSE16515 and GSE28735 datasets in the GEO database also indicated that the transcriptional level of PSMD14 is relatively high in tumor tissues (Fig. 2B). More importantly, the overall survival and progression-free survival of PC patients with high expression of PSMD14 were significantly lower than those with low expression of PSMD14, according to analysis of the survival data from the TCGA-PAAD dataset (Fig. 2C). Although the chi-square analysis indicated that only the differentiation level had statistical differences among patients with different expression levels of PSMD14 in the analyzed clinical features (Table 1), the results still showed that the overall expression level of PSMD14 in tumor tissues was greater than that in nontumor tissues, and high expression of PSMD14 was a risk factor for a relatively poor prognosis, in conjunction with IHC, log-rank test and COX proportional hazards model (Fig. 2D–G). To investigate the potential mechanism by which PSMD14 leads to poor prognosis, pathway enrichment analysis was conducted based on the TCGA-PAAD dataset. In addition to regulating the WNT pathway, PSMD14 may also be involved in glycolysis, pyrimidine metabolism, mismatch repair, and base excision repair, pathways related to glycolysis and gemcitabine resistance (Fig. 2H).

**Table 1:** The relationship between PSMD14 expression and PC clinicopathologic features.

Clinicopathologic feature	PSMD14 expression		*p*
	Low	High	
All cases	45	45	
Age			
<60	23	22	0.8330
≥60	22	23	
Gender			
Male	26	26	1
Female	19	19	
Differentiation			
Moderately and well	40	30	0.0112
Poorly	5	15	
AJCC stage			
Ⅰ	16	18	0.6637
Ⅱ, Ⅲ, and Ⅳ	29	27	

### PSMD14 promotes PC glycolysis and induces insensitivity to gemcitabine

**Fig. 3:**
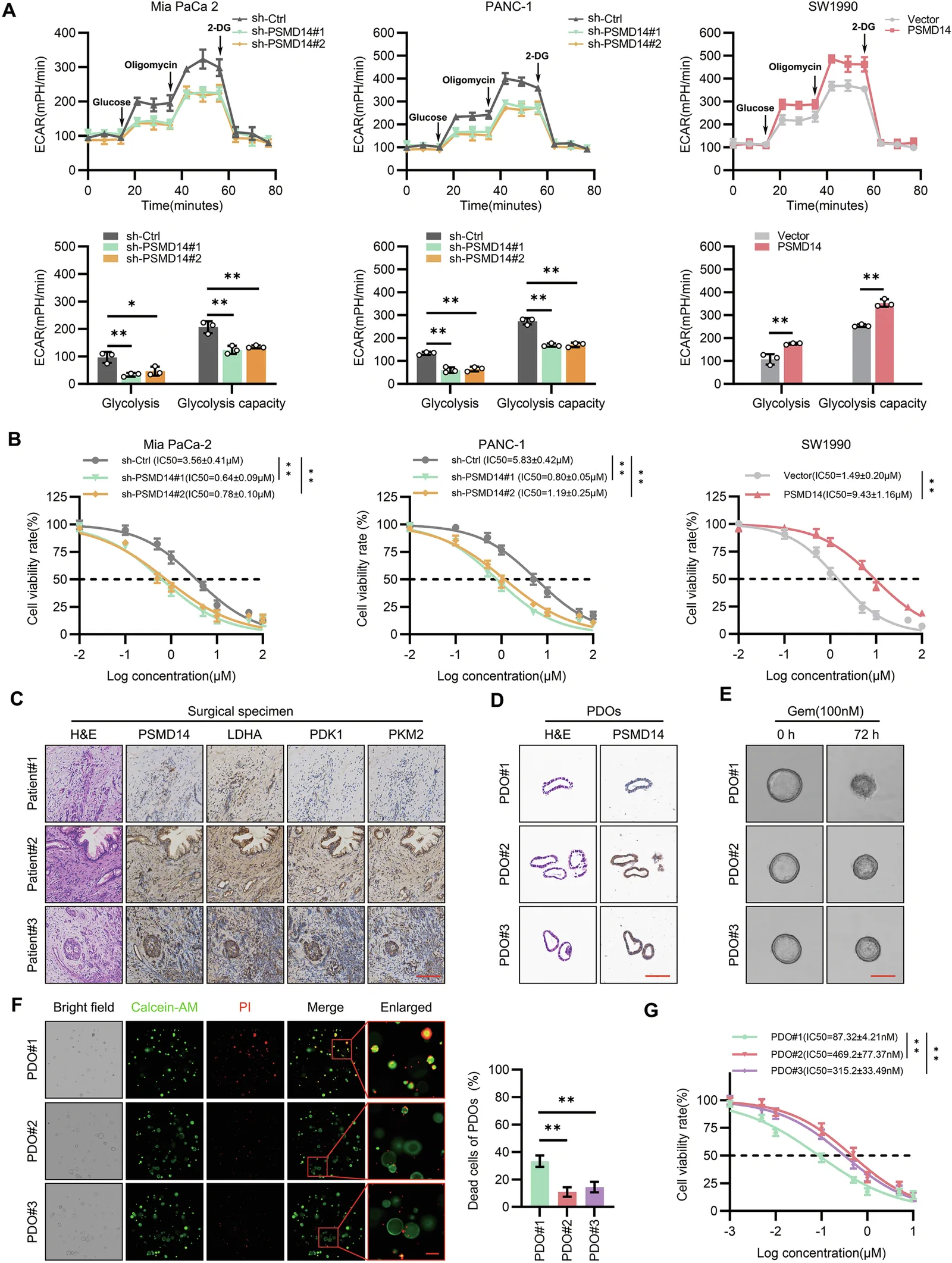
PSMD14 promotes pancreatic cancer glycolysis and induces insensitivity to gemcitabine. **A** The effect of PSMD14 on the glycolytic level of pancreatic cancer cells was detected via the ECAR (*n* = 3). **B** The effect of PSMD14 on the sensitivity of pancreatic cancer cells to gemcitabine was detected via a cell viability assay (*n* = 5). **C** Representative images showing the expression of PSMD14 and glycolysis-related proteins in clinical samples; scale bar = 200 μm. **D** The expression of PSMD14 in patient-derived organoids was detected by IHC; scale bar = 100 μm. **E** PDO response to gemcitabine (100 nM for 72 h) was detected by microscopy; scale bar = 100 μm. **F** PDO response to gemcitabine (100 nM for 72 h) was detected by Calcein-AM/PI, scale bar = 100 μm, (*n* = 6). **G** The effect of PSMD14 on the sensitivity of PDOs to gemcitabine was detected via a cell viability assay (*n* = 5). *P < 0.05, **P < 0.01.

To determine whether PSMD14 influences glycolysis and gemcitabine sensitivity in PC, ECAR, glucose uptake, L-lactate production, gemcitabine sensitivity, γ-H2AX staining assays, and expression detection of enzymes involved in gemcitabine metabolism, a public database analysis was conducted. The results revealed that PSMD14 is up-regulated in some gemcitabine-resistant pancreatic cancer cells and knockdown of PSMD14 significantly inhibited glycolysis and effectively reduced the IC50 of gemcitabine, whereas the overexpression of PSMD14 had the opposite effect (Figs. 3A, B and S2A–E). Several downstream target genes of the WNT/β-catenin signaling pathway related to glycolysis were subsequently detected via RT‒qPCR [17, 18]. Among these glycolysis-related genes (GRGs), LDHA, PDK1, and PKM2 were significantly affected by the abundance of PSMD14 expression, and the transcriptional level correlations between them and PSMD14 were analyzed via the TCGA and GTEx databases (Fig. S2F, G). Moreover, their protein expression levels were also consistent with those of PSMD14 (Fig. S2H). Furthermore, the expression levels of LDHA, PDK1, PKM2, and PSMD14 in clinicopathological tissues were also detected, and the results also revealed that PSMD14 was positively correlated with the protein expression levels of these key glycolysis genes (Fig. 3C). The patient-derived organoids were isolated, cultured, and expanded from tumor tissues of pancreatic cancer from the corresponding tumor tissues. The IHC results revealed that the organoids retained the characteristics of the PSMD14 expression level in corresponding tumor tissues (Fig. 3D). More importantly, when the organoids with relatively low PSMD14 expression were treated with gemcitabine, organoid disruption was more pronounced, more cells died, and the cell viability was lower (Fig. 3E–G). Treating with 2-DG or interfering with LDHA expression in PC cells to suppress glycolysis levels also significantly increased cell sensitivity to gemcitabine (Fig. S2I).

### Inhibition of the WNT/β-catenin signaling pathway restricts the function of PSMD14

**Fig. 4:**
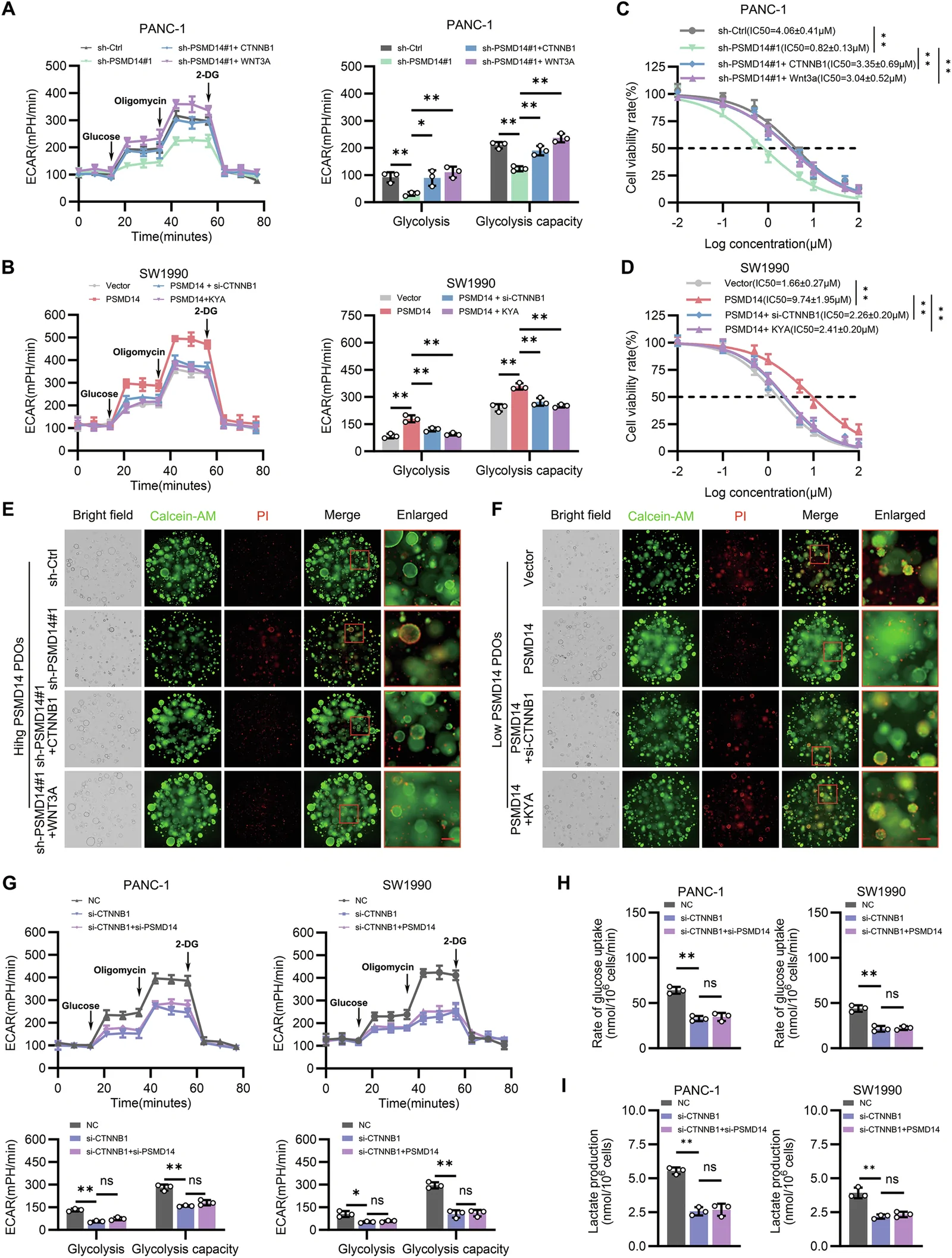
Inhibition of the WNT/β-catenin signaling pathway restricts the function of PSMD14. **A** Effects of transient transfection with CTNNB1 or treatment with WNT3A in PSMD14-knockdown PANC-1 cells on glycolysis were detected by ECAR (*n* = 3). **B** Effects of transient transfection with si-CTNNB1 or treatment with KYA in PSMD14-knockin SW1990 cells on glycolysis were detected by ECAR (*n* = 3). **C** The effect of transient transfection of PSMD14-knockdown PANC-1 cells with CTNNB1 or WNT3A on sensitivity to gemcitabine was detected via a cell viability assay (*n* = 5). **D** The effect of transient transfection of si-CTNNB1 or KYA on the sensitivity of PSMD14-knockdown SW1990 cells to gemcitabine was detected via a cell viability assay (*n* = 5). **E** Effects of transient transfection of PSMD14-knockdown PDOs with CTNNB1 or WNT3A on sensitivity to gemcitabine were detected via Calcein-AM/PI; scale bar = 100 μm. **F** The effect of transient transfection of si-CTNNB1 or KYA on the sensitivity of PDOs to gemcitabine was detected by Calcein-AM/PI, scale bar = 100 μm. **G** Effects of transient transfection with si-PSMD14 or PSMD14 in CTNNB1-interfered PC cells on glycolysis were detected by ECAR (*n* = 3). **H** Effects of transient transfection with si-PSMD14 or PSMD14 in CTNNB1-interfered PC cells on glycolysis were detected by glucose uptake (*n* = 3). **I** Effects of transient transfection with si-PSMD14 or PSMD14 in CTNNB1-interfered PC cells on glycolysis were detected by lactate production (*n* = 3). ns means not significant, *P < 0.05, **P < 0.01.

To verify whether the above regulatory effects of PSMD14 are achieved through the regulation of the WNT/β-catenin signaling pathway, corresponding rescue experiments were performed. First, ECAR, glucose uptake, and L-lactate production assays revealed that activation of the WNT/β-catenin signaling pathway reversed the inhibitory effect of PSMD14 knockdown on glycolysis in PC cells (Figs. 4A and S3A). Accordingly, the catalytic effect of PSMD14 on glycolysis was limited by genetic or pharmacologic inhibition of WNT/β-catenin pathway activity (Figs. 4B and S3B).

Moreover, the results of gemcitabine sensitivity tests and Calcein-AM/PI double-stain experiments revealed that the overexpression of PSMD14 reduced the sensitivity of tumor cells to gemcitabine. However, the increased sensitivity of tumor cells to gemcitabine caused by PSMD14 knockdown was limited by genetic or pharmacologic activation of the WNT/β-catenin signaling pathway. Correspondingly, genetic or pharmacologic inhibition of the WNT/β-catenin signaling pathway restored tumor cell sensitivity to gemcitabine (Figs. 4C–F and S3D–F).

Lastly, various experimental results, including ECAR, glucose uptake, and lactate production, showed that in pancreatic cancer cells with CTNNB1 interference, the effects of interfering with or overexpressing PSMD14 on glycolysis levels were not statistically significant (Fig. 4G–I).

### PSMD14 removes the K63-linked ubiquitin chain of DVL1 and facilitates its membrane trafficking

**Fig. 5:**
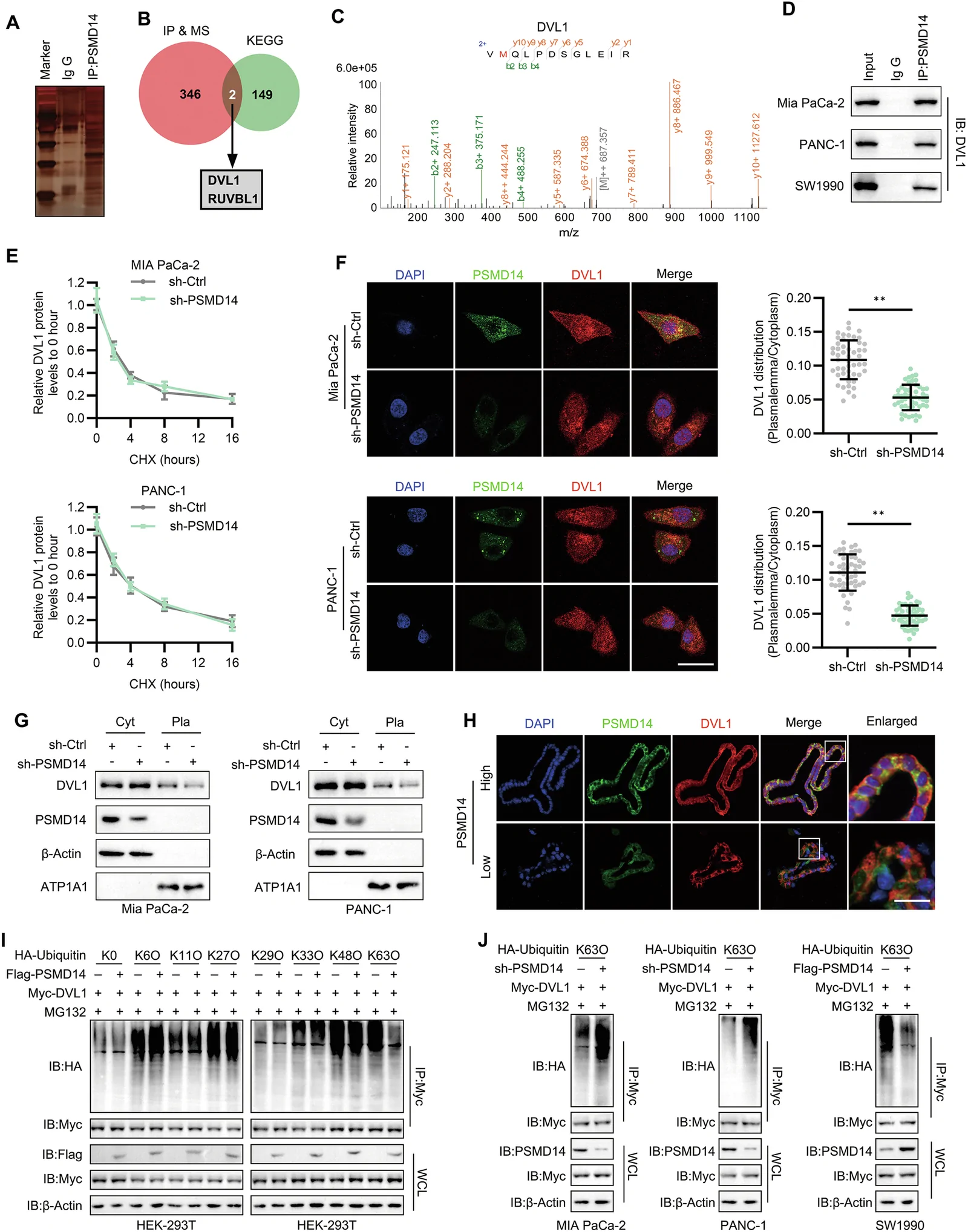
PSMD14 removes the K63-linked ubiquitin chain of DVL1 and facilitates its membrane trafficking. **A** Immunoprecipitation was carried out using silver staining. **B** Venn diagram of proteins that interact with PSMD14 identified by mass spectrometry and key genes of the WNT pathway. **C** The secondary spectrum of DVL1. **D** The interactions between PSMD14 and DVL1 in different cells were detected by IP. **E** Relative quantitative statistics of the DVL1 protein after cycloheximide treatment (50 μM) (*n* = 3). **F** Representative images and statistical graphs of the distribution of the DVL1 protein within pancreatic cancer cells; scale bar = 20 μm, *n* = 50. **G** The distribution of DVL1 protein within the cytoplasm (Cyt) and plasmalemma (Pla) was detected by western blotting. **H** The distribution of the DVL1 protein within the plasmalemma and cytoplasm of PDOs with different PSMD14 abundances was detected by mIHC; scale bar = 25 μm. **I** The influence of PSMD14 on various types of ubiquitin chain linkages of DVL1 was identified in 293T cells. **J** The influence of PSMD14 on the K63 ubiquitin chain linkage of DVL1 was identified in pancreatic cancer cells. ns means not significant, *P < 0.05, **P < 0.01.

Coimmunoprecipitation and mass spectrometry were then used to identify potential proteins that interact with PSMD14 in PC cells, thereby revealing the molecular mechanisms by which PSMD14 regulates the WNT/β-catenin signaling pathway. A total of 348 protein molecules that may interact with PSMD14 were identified in PANC-1 cell lysates, among which DVL1 and RUVBL1 are considered to be closely related to WNT/β-catenin signaling pathway activity (Fig. 5A–C). The interaction between DVL1 and PSMD14 was subsequently confirmed in different pancreatic cell lines, whereas the interaction between RUVBL1 and PSMD14 was not confirmed (Figs. 5D and S4A). However, the expression and stability of DVL1 in PC cells did not change significantly with changes in PSMD14 (Figs. 5E and S4B). Immunofluorescence and western blot results revealed that the distribution of DVL1 in the plasmalemma (Pla) was reduced when PSMD14 was knocked down (Fig. 5F, G). In organoids with relatively high expression of PSMD14, the distribution of DVL1 in the plasmalemma was also greater (Fig. 5H). Moreover, to determine which type of ubiquitin chain linkage(s) in cells is affected by the expression level of PSMD14, we cotransfected DVL1 with 8 ubiquitin K-only constructs. The 8 ubiquitin K-only constructs had all lysine residues mutated to arginines (K0) or only one indicated lysine residue, while the remaining 6 lysine residues were mutated to arginines (K6O, K11O, K27O, K29O, K33O, K48O, and K63O). The results revealed that only when HEK-293T cells were cotransfected with DVL1 and K63O constructs did the overexpression of PSMD14 significantly reduce the level of ubiquitinated DVL1 (Fig. 5I). Subsequent experiments in PC tumor cell lines further confirmed that PSMD14 removes the K63-linked ubiquitin chain of DVL1 (Fig. 5J).

### PSMD14 removes the K63-linked ubiquitin chain of DVL1 at the K486 residue

**Fig. 6:**
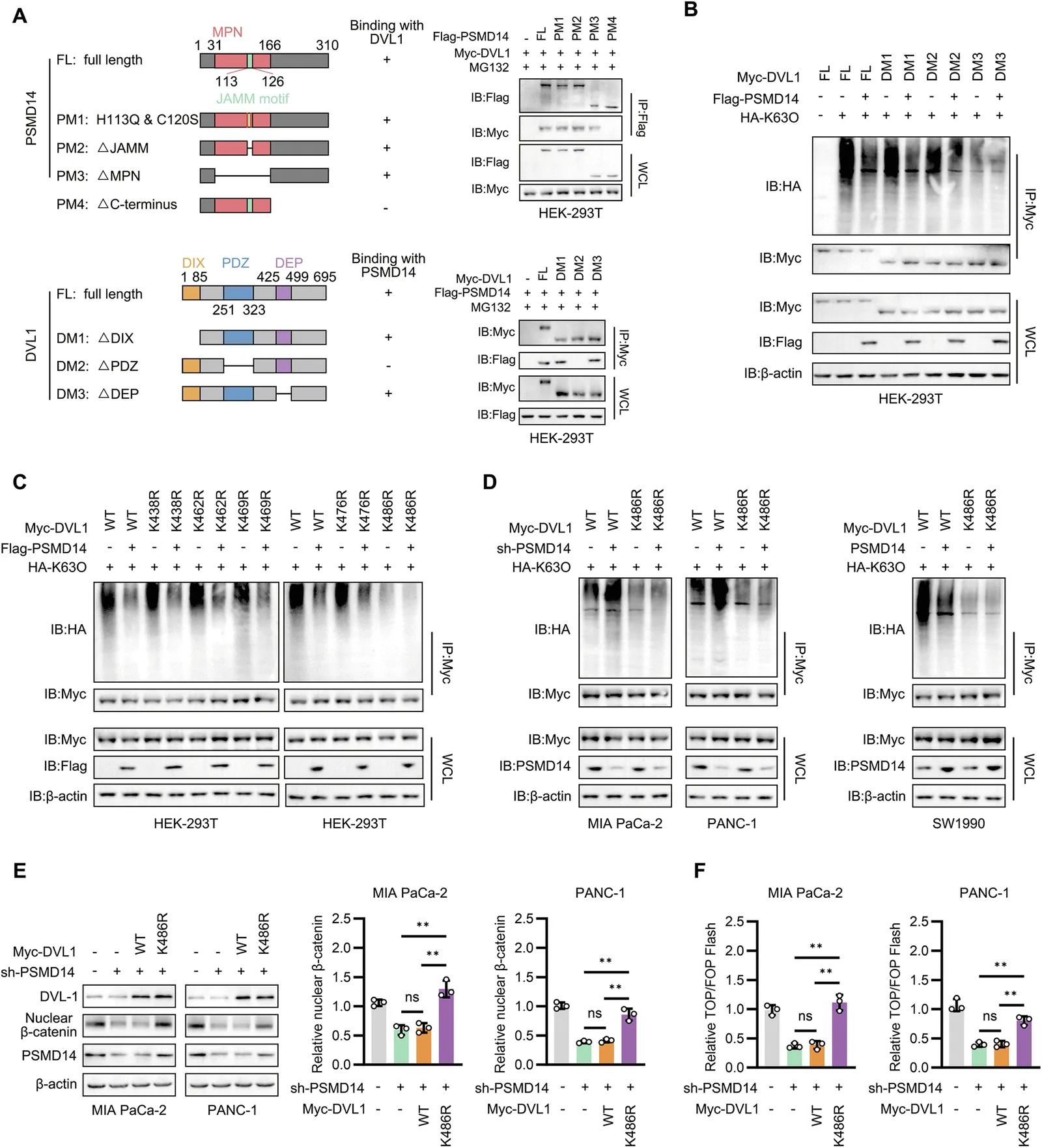
PSMD14 removes the K63-linked ubiquitin chain of DVL1 at the K486 residue. **A** A variety of mutant and truncated forms of PSMD14 or DVL1 were constructed for analyzing the interaction segments between PSMD14 and DVL1 in 293T cells. **B** Various types of truncations of DVL1 were constructed to analyze whether PSMD14 mainly removes the K63-linked ubiquitin chain, which is the domain of DVL1 in 293T cells. **C** Mutant constructs of lysine sites in the DEP domain were constructed to detect the K63-linked ubiquitin chain, from which the lysine site was removed by PSMD14. **D** After the K486 site of DVL1 was mutated, the ability of PSMD14 to remove the K63-linked ubiquitin chain of DVL1 was detected in pancreatic cancer. **E** Nuclear β-catenin expression was detected when WT or K486R DVL1 plasmids were introduced into PC cells (*n* = 3). **F** TOP/FOP flash was conducted when WT or K486R DVL1 plasmids were introduced into PC cells (*n* = 3). ns means not significant, *P < 0.05, **P < 0.01.

Furthermore, to identify the specific sites where PSMD14 plays a role, a variety of mutants and truncations were constructed. In HEK-293T cells, the loss-of-function mutation of the deubiquitination enzyme C120S and H113Q and the deletion of the JAMM motif or even the domain of the MPN did not hinder the interaction between PSMD14 and DVL1. However, when the C-terminus of PSMD14 was removed, the interaction between PSMD14 and DVL1 disappeared. Moreover, the deletion of the PDZ domain of DVL1 caused the interaction between PSMD14 and DVL1 to disappear (Fig. 6A).

On the other hand, removing the DIX or PDZ domain of DVL1 did not prevent PSMD14 from removing the K63-linked ubiquitin chain of DVL1. After the DEP domain of DVL1 was deleted, the K63-linked ubiquitination level of DVL1 significantly decreased, and the deubiquitination effect of PSMD14 on DVL1 disappeared (Fig. 6B). The DEP domain of DVL1 contains 5 lysine sites that can undergo ubiquitination. After each lysine site was mutated to arginine without ubiquitination, we found that K486R significantly reduced the K63-linked ubiquitination of DVL1 and that K486R interfered with the deubiquitination of PSMD14 on DVL1 (Fig. 6C). The same results were confirmed in PC cells (Fig. 6D).

Meanwhile, transfection of the K486R mutant-type instead of the wild-type DVL1 plasmid in PSMD14 knockdown PC cells could reverse the inhibition of PSMD14 knockdown on nuclear β-catenin expression and WNT/β-catenin pathway transcriptional activity (Fig. 6E, F).

Together, these results suggest that PSMD14 interacts with the PDZ domain of DVL1 through its C-terminus and removes the K63-linked ubiquitin chain of DVL1 at the K486 site.

### Activation of the WNT/β-catenin pathway promotes PSMD14 transcription

**Fig. 7:**
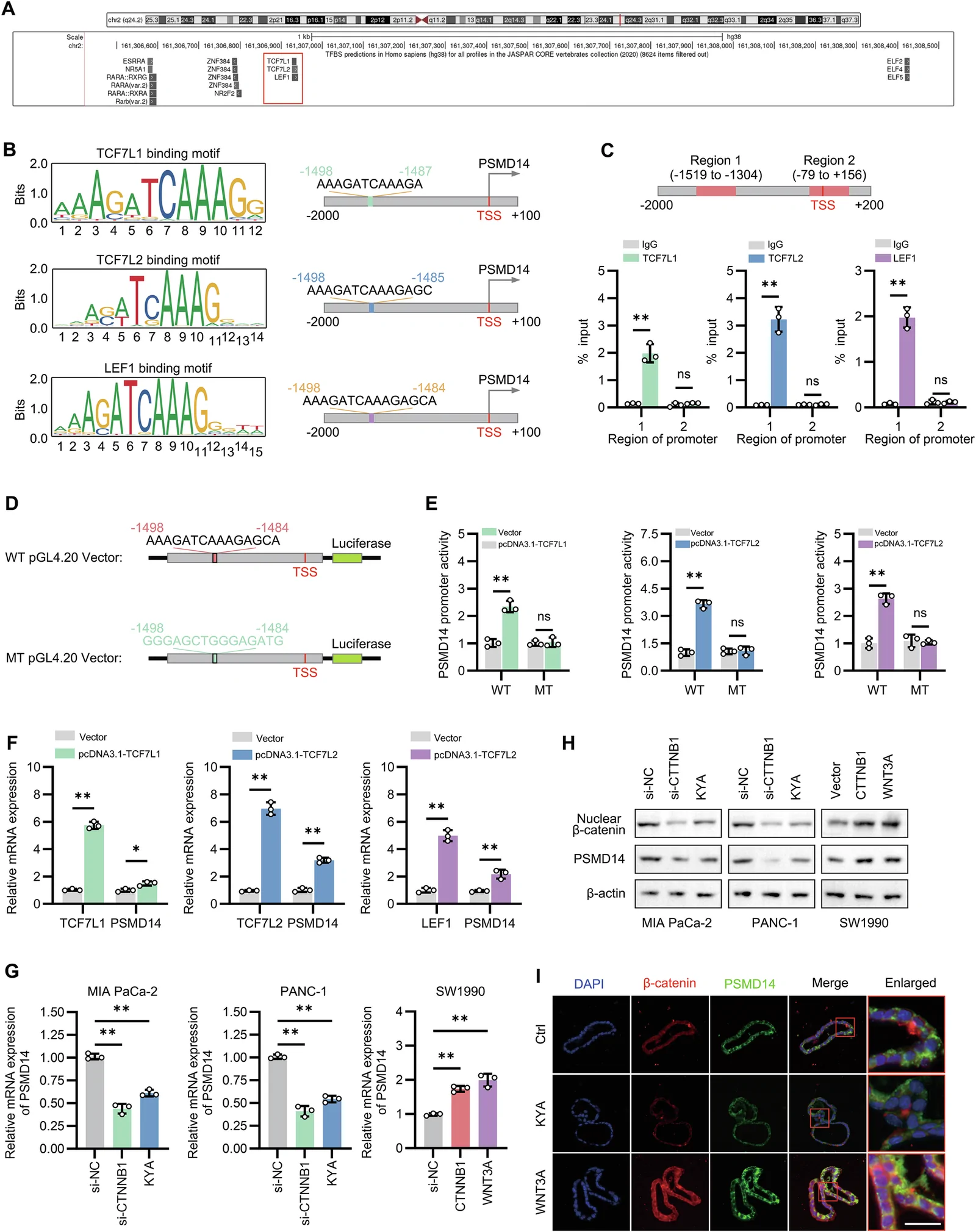
Activation of the WNT/β-catenin pathway promotes PSMD14 transcription. **A** The transcription factors that can bind to the promoter region of PSMD14 were predicted using the UCSC database. **B** The binding sequences of the transcription factors TCF7L1, TCF7L2, and LEF1 (left panel), as well as the potential binding site patterns of these transcription factors in the promoter region of PSMD14 (right panel). **C** Whether the transcription factors TCF7L1, TCF7L2, and LEF1 can interact with the promoter region of PSMD14 was detected via ChIP‒PCR (*n* = 3). **D** Promoter sequences containing the wild-type (WT) and mutant (MT) binding sites were inserted into the pGL4.20 vector. **E** A dual-luciferase reporter gene assay was used to detect alterations in the transcriptional activity of the transcription factor after the mutation binding sequence was introduced (*n* = 3). **F** The mRNA of PSMD14 was detected after TCF7L1, TCF7L2, or LEF1 plasmids were introduced in PANC-1 cells (*n* = 3). **G** RT‒qPCR was used to detect changes in the transcriptional level of PSMD14 after inhibition or activation of the WNT pathway (*n* = 3). **H** Western blot was used to detect changes in the translation level of PSMD14 after inhibition or activation of the WNT pathway. **I** mIHC was used to detect changes in the translation level of PSMD14 in PDOs after inhibition or activation of the WNT pathway. scale bar = 25 μm. ns not significant, *P < 0.05, **P < 0.01.

Thus, what mechanism leads to the increase in PSMD14 transcription levels in various digestive system malignancies? When the promoter region of PSMD14 was analyzed with the UCSC database, sequences were found to be potential binding sites for the TCF7L1, TCF7L2, and LEF1 transcription factors (Fig. 7A). When the WNT/β-catenin signaling pathway is activated, β-catenin enters the nucleus and interacts with LEF/TCF family transcription factors, including TCF7L1, TCF7L2, and LEF1, to regulate downstream gene transcription. Therefore, we examined whether the increase in PSMD14 expression in PC is caused by abnormal activation of the WNT/β-catenin signaling pathway, that is, whether the increase in PSMD14 expression and abnormal activation of the WNT/β-catenin signaling pathway form a positive feedback loop. The predicted binding sites of the TCF7L1, TCF7L2, and LEF1 transcription factors and their potential binding sequences in the PSMD14 promoter region are shown in Fig. 7B. Subsequently, ChIP experiments with PANC-1 cells revealed that TCF7L1, TCF7L2, and LEF1 did indeed interact with the predicted binding sequence (Fig. 7C). Moreover, to further determine whether the transcription of PSMD14 is regulated by these three transcription factors, promoter sequences containing wild-type (WT) or mutant (MT) binding sites were cloned and inserted into the pGL4.20 plasmid and transfected into PC cells via the corresponding plasmids. The results revealed that the transcriptional activity of PSMD14 increased when TCF7L1, TCF7L2, and LEF1 were cotransfected with the wild-type binding site, but there were no significant changes when TCF7L1, TCF7L2, and LEF1 were cotransfected with the mutant binding site (Fig. 7D, E). When TCF7L1, TCF7L2, and LEF1 overexpression plasmids were introduced into PANC-1 cells, the expression of PSMD14 was also up-regulated (Fig. 7F). The data analysis results of the TCGA and GTEx databases showed that the transcriptional level of PSMD14 was positively correlated with TCF7L1, TCF7L2, and LEF1 (Fig. S5A). In addition, the results of RT‒qPCR, western blot, and mIHC confirmed that the expression level of PSMD14 was regulated by the activity of the WNT/β-catenin signaling pathway (Fig. 7G‒I). These results suggest that the transcription of PSMD14 is facilitated by the activation of the WNT/β-catenin signaling pathway. The transcription of PSMD14 and the abnormal activation of the β-catenin signaling pathway form a positive feedback loop.

### Targeting PSMD14 sensitizes PC to gemcitabine

**Fig. 8:**
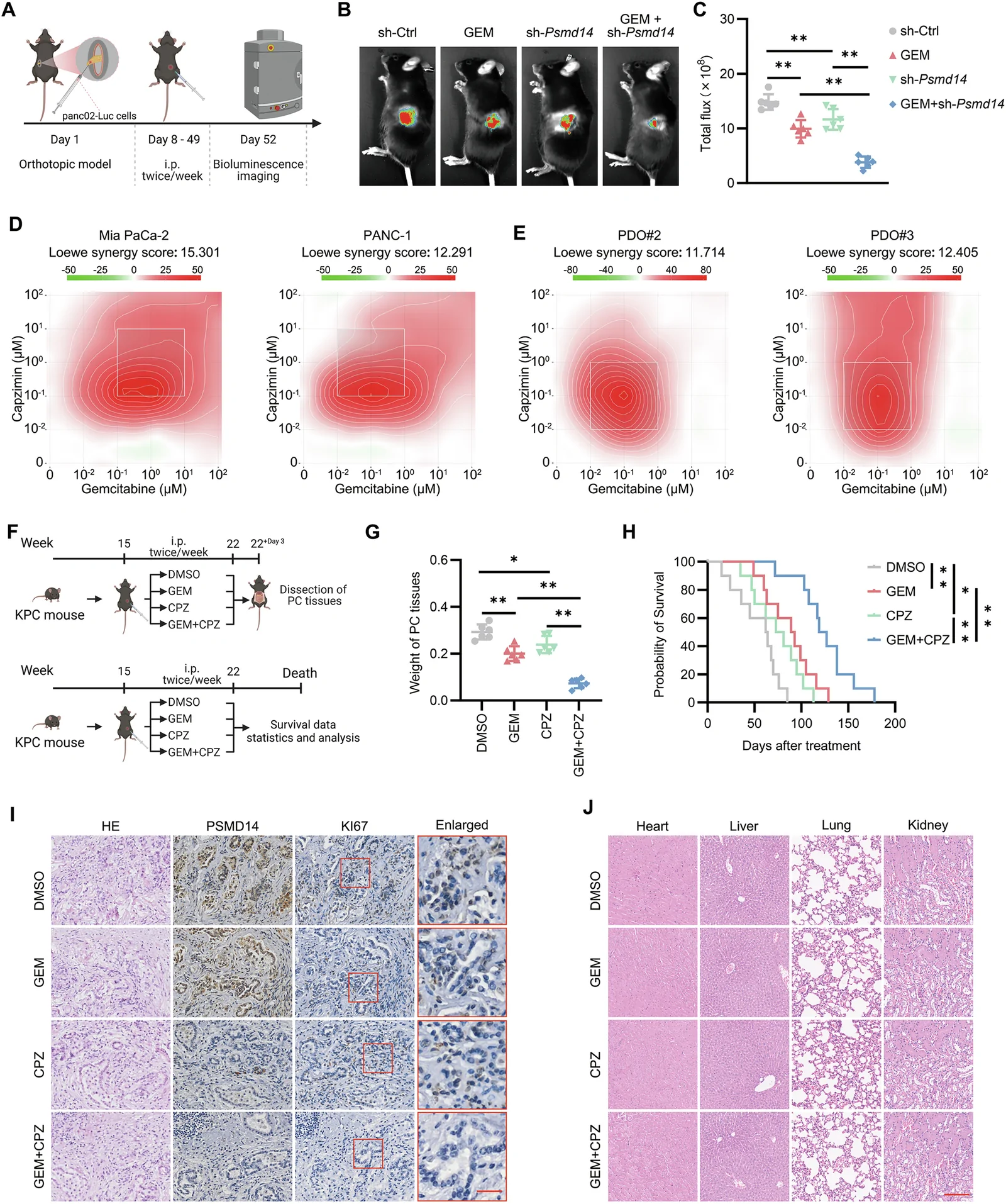
Targeting PSMD14 sensitizes pancreatic cancer to gemcitabine. **A** Experimental model of C57BL/6 mice. **B** Representative images of in vivo imaging of C57BL/6 mice. **C** Statistical graphs of in vivo imaging for each group of C57BL/6 mice (*n* = 6). **D** Synergy analysis of gemcitabine and capzimin using the Loewe model in MIA PaCa-2 and PANC-1 cells. **E** Synergy analysis of gemcitabine and capzimin in PDOs via the Loewe model. **F** Experimental model of KPC mice. **G** Tumor weights of pancreatic tissues from KPC mice (*n* = 6). **H** Overall survival analysis of KPC mice (*n* = 10). **I** The expression of PSMD14 and Ki67 in the pancreatic cancer tissues of each group of KPC mice was detected by IHC; scale bar = 50 μm. **J** The hearts, livers, lungs, and kidneys of each group of KPC mice were examined using HE staining. scale bar = 200 μm. ns means not significant, *P < 0.05, **P < 0.01.

Finally, to further clarify whether targeting PSMD14 affects the gemcitabine sensitivity of PC cells in vivo, we conducted relevant studies. First, Panc02 cells from each group were injected into the pancreata of the mice, and gemcitabine or its control solvent was injected intraperitoneally 7 days later. At the end of the experiment, in vivo imaging was performed to detect tumor cell proliferation in each group. The results revealed that the inhibitory effect of gemcitabine on Panc02 cell proliferation was enhanced after the expression of PSMD14 was decreased (Fig. 8A–C). Subsequently, PC cells and patient-derived PC organoid models were treated with a combination of gemcitabine and capzimin at different concentrations, and the results were analyzed using the Loewe additivity method. The results showed that gemcitabine and capzimin had a significant synergistic effect on inhibiting the viability of tumor cells and patient-derived PC organoid cells (Fig. 8D, E).

Subsequently, experiments were conducted to evaluate whether CPZ had a significant impact on cell viability and proteasome function. Calcein-AM/PI results showed that, when pancreatic cancer cells were treated with CPZ alone (100 μM) for 24 h, there was no significant difference in cell activity between the DMSO group and CPZ group (Fig. S6A). Meanwhile, proteasome activity assay indicated that knockdown of PSMD14 or CPZ (100 μM for 24 h) had no significant effect on the proteasome function of PC cells (Fig. S6B). What is more, when PC cells were treated with the proteasome inhibitor MG132 (10 μM for 24 h), it showed a promoting effect on glycolysis of pancreatic cancer cells rather than an inhibitory effect like CPZ (Fig. S6C–E). These results together suggest that the effect of PSMD14 inhibition on the glycolysis level of pancreatic cancer cells, whether genetic or pharmacological, does not seem to be achieved by affecting the overall function of the proteasome.

Finally, we intraperitoneally injected gemcitabine and capzimin into KPC model mice to evaluate tumor growth and survival, respectively. The results showed that gemcitabine and capzimin combined with intraperitoneal injection effectively inhibited the progression of PC in mice and prolonged their survival (Fig. 8F–H). In addition, HE staining and IHC experiments further confirmed that gemcitabine combined with capzimin reduced the positive rate of Ki67 in the PC tissues of KPC mice, and the combination did not cause significant damage to the heart, liver, lung, or kidney of the mice (Fig. 8I, J).

## Discussion

Abnormal activation of the WNT/β-catenin signaling pathway promotes the progression and therapy resistance of multiple malignancies [19]. Hence, the WNT/β-catenin signaling pathway has become a very attractive therapeutic target in recent years [20–22]. Further elucidation of the mechanisms underlying the abnormal activation of WNT/β-catenin signaling in PC could help improve the overall effectiveness of current therapies. Moreover, deubiquitinating enzymes are proteins that are able to influence the stability, intracellular distribution, and function of protein substrates by influencing the type and intensity of ubiquitination modifications, and thus have a significant impact on cell signal transduction [23]. Therefore, searching for deubiquitination enzymes that regulate WNT/β-catenin signaling in tumors is highly important.

In this study, we screened and verified that PSMD14, a deubiquitinating enzyme that functions in the malignant progression and treatment resistance of tumors, significantly affects WNT/β-catenin signaling pathway activity in PC and that its high expression status is a risk factor for poor prognosis with PC [24–28]. These findings suggest that PSMD14 is a promising potential therapeutic target for PC.

PSMD14 has a significant effect on different types of ubiquitin-linked chains, thereby exerting a significant distinct influence on cellular functions [25, 29–31]. This study revealed that the intracellular distribution of PSMD14 in PC cells is affected by the removal of the K63-linked ubiquitin chain in the DEP region of DVL1. DVL1 belongs to the disheveled family, which is an important regulator involved in the WNT/β-catenin signaling pathway [32, 33]. Ubiquitination of DVL1 can weaken its ability to bind phosphatidic acid, prevent its transposition to the cytomembrane, and reduce its ability to recruit β-catenin destruction complex components to the cytomembrane; as a result, β-catenin accumulates, enters the nucleus, and binds to TCF/LEF to increase the transcription of glycolysis-related genes [34, 35]. These results reveal an important mechanism by which PSMD14 regulates WNT/β-catenin signaling in PC.

Since enhanced glycolysis increases glucose uptake by tumor cells, pyrimidine biosynthesis increases intrinsic levels of deoxycytidine triphosphate and reduces effective levels of gemcitabine through molecular competition, and glycolysis is believed to be closely associated with resistance to gemcitabine chemotherapy [36, 37]. In this study, we confirmed that PSMD14 enhances glycolysis in PC and limits tumor sensitivity to gemcitabine. Moreover, this change may be realized through the activation of the WNT/β-catenin signaling pathway by PSMD14 to promote the transcription of well-known glycolytic-related enzymes such as LDHA, PDK1, and PKM2 [17]. In addition, PSMD14 transcription can also be promoted by the activation of the WNT/β-catenin signaling pathway. These results indicate a positive feedback loop between PSMD14 expression and WNT/β-catenin signaling pathway activity, revealing the cause of the abnormally high expression of PSMD14 in PC and the potential mechanism by which high expression of PSMD14 leads to resistance to gemcitabine therapy in PC.

Given their critical regulatory role in the development and progression of malignant tumors, various inhibitors of the WNT pathway have been identified and developed in the quest to treat cancer. However, these inhibitors usually have insufficient specificity and inevitable toxic side effects, which hinder their clinical application [38]. The preliminary results of this study showed that targeting PSMD14 may be a potential strategy for inhibiting abnormal activation of the WNT/β-catenin signaling pathway in PC, reducing the level of glycolysis in tumor cells, and increasing the therapeutic effect of gemcitabine. Interestingly, both in vivo and in vitro experimental results have shown that capzimin, a widely used PSMD14 inhibitor, can achieve these effects without causing significant organ damage [39, 40]. These results suggest that targeting PSMD14 may be an effective way to increase the sensitivity of PC to gemcitabine, improve prognosis, and improve survival time.

Undeniably, current research also has certain limitations. On the one hand, the ubiquitin proteasome system is the most important ATP-dependent degradation system related to protein quality control and cell homeostasis maintenance in eukaryotic cells. In this system, the 26S proteasome consists of a 20S core particle and a 19S regulatory particle, and PSMD14 serves as one of the 19 essential subunits of the complete assembled 19S regulatory particle. Genetic or pharmacological intervention in the expression or function of PSMD14 may lead to changes in the ubiquitination modification levels of a large number of proteins, not just DVL1. On the other hand, the change of PSMD14 in pancreatic cancer may not only affect the single WNT/β-catenin signaling, but also involve the change of activity and metabolic level of other pathways. More in-depth follow-up research is still needed to further clarify the role of PSMD14 in the occurrence and development of PC.

**Fig. 9:**
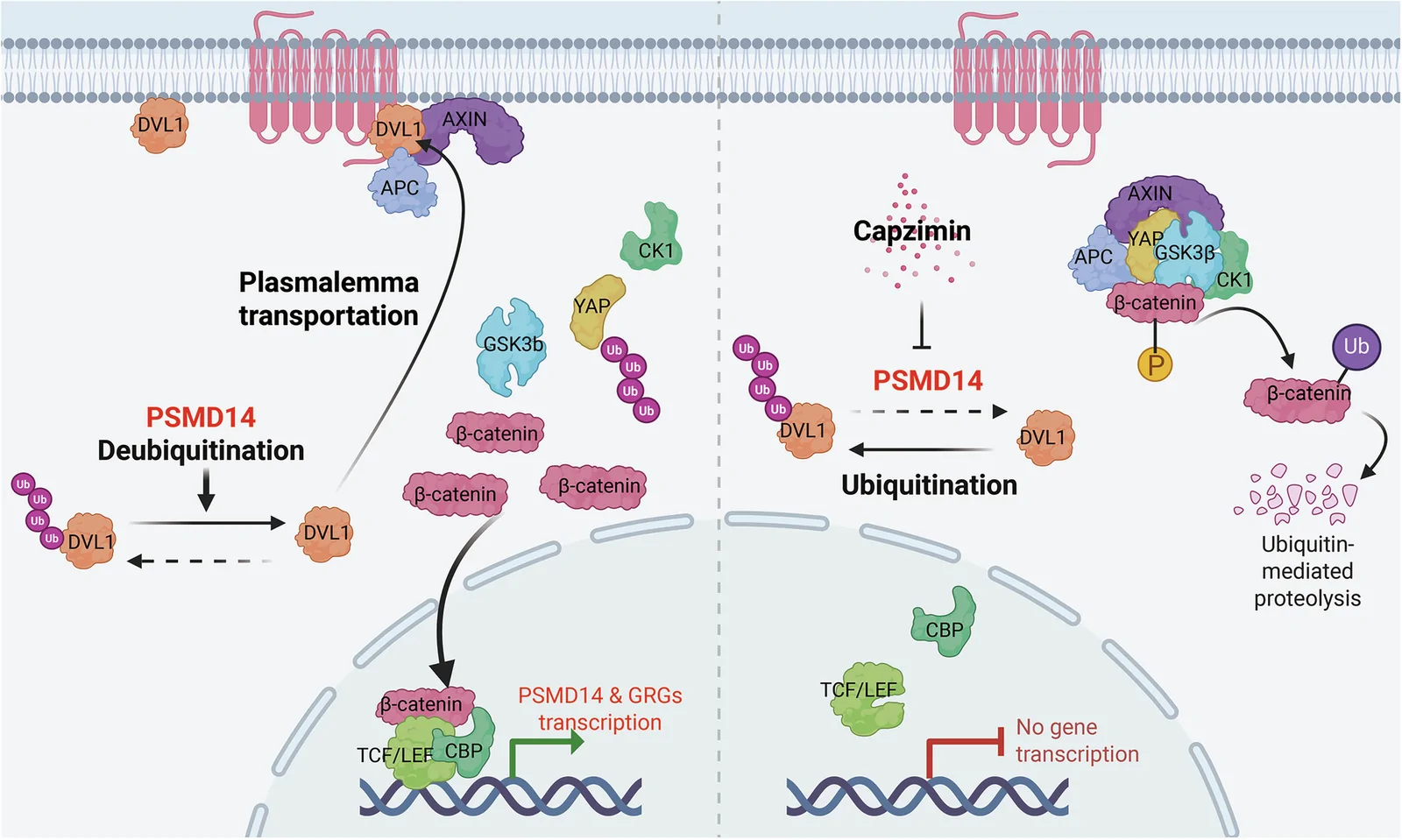
Schematic diagram of this study, created with BioRender.com (Agreement number: VO299EXWL4).

In conclusion, this study identified PSMD14, a key deubiquitination enzyme that induces abnormal activation of the WNT pathway in PC. By removing the K63-linked ubiquitin chain of DVL1, PSMD14 activated the WNT/β-catenin signaling pathway, promoting the transcription of glycolysis-related genes and PSMD14, enhancing glycolysis in PC and reducing sensitivity to gemcitabine chemotherapy. Capzimin restricts the activity of the abnormally activated WNT/β-catenin signaling pathway by inhibiting the function of PSMD14, increasing sensitivity to gemcitabine chemotherapy, and limiting the progression of PC (Fig. 9). These findings offer a promising therapeutic strategy for improving the antitumor efficacy and prognosis of PC.

## Supplementary information

Supplemental materials

Full and uncropped western blots

## Data Availability

The data supporting the findings of this study are available from the corresponding author upon reasonable request.

## References

[1] Siegel RL, Giaquinto AN, Jemal A. Cancer statistics, 2024. CA: A Cancer J Clin. 2024;74:12–49. 10.3322/caac.21820 38230766

[2] Halbrook CJ, Lyssiotis CA, Pasca di Magliano M, Maitra A. Pancreatic cancer: advances and challenges. Cell. 2023;186:1729–54. 10.1016/j.cell.2023.02.014 PMC10182830 37059070

[3] Del Chiaro M, Sugawara T, Karam SD, Messersmith WA. Advances in the management of pancreatic cancer. BMJ. 2023;383:e073995. 10.1136/bmj-2022-073995 38164628

[4] Gyawali B, Booth CM. Treatment of metastatic pancreatic cancer: 25 years of innovation with little progress for patients. Lancet Oncol. 2024;25:167–70. 10.1016/S1470-2045(23)00516-8 38301687

[5] Nevala-Plagemann C, Garrido-Laguna I. NALIRIFOX for metastatic pancreatic adenocarcinoma: hope or hype? Nat Rev Clin Oncol. 2024;21:567–8. 10.1038/s41571-024-00896-w 38664532

[6] Mastrantoni L, Chiaravalli M, Spring A, Beccia V, Di Bello A, Bagala C, et al. Comparison of first-line chemotherapy regimens in unresectable locally advanced or metastatic pancreatic cancer: a systematic review and Bayesian network meta-analysis. Lancet Oncol. 2024;25:1655–65. 10.1016/S1470-2045(24)00511-4 39542008

[7] Liu J, Xiao Q, Xiao J, Niu C, Li Y, Zhang X, et al. Wnt/beta-catenin signalling: function, biological mechanisms, and therapeutic opportunities. Signal Transduct Target Ther. 2022;7:3. 10.1038/s41392-021-00762-6 PMC8724284 34980884

[8] Nusse R, Clevers H. Wnt/beta-catenin signaling, disease, and emerging therapeutic modalities. Cell. 2017;169:985–99. 10.1016/j.cell.2017.05.016 28575679

[9] Zhou C, Zhu X, Liu N, Dong X, Zhang X, Huang H, et al. B-lymphoid tyrosine kinase-mediated FAM83A phosphorylation elevates pancreatic tumorigenesis through interacting with beta-catenin. Signal Transduct Target Ther. 2023;8:66. 10.1038/s41392-022-01268-5 PMC9935901 36797256

[10] Kim MP, Li X, Deng J, Zhang Y, Dai B, Allton KL, et al. Oncogenic KRAS recruits an expansive transcriptional network through mutant p53 to drive pancreatic cancer metastasis. Cancer Discov. 2021;11:2094–111. 10.1158/2159-8290.CD-20-1228 PMC8338884 33839689

[11] Zheng S, Lin J, Pang Z, Zhang H, Wang Y, Ma L, et al. Aberrant cholesterol metabolism and Wnt/beta-catenin signaling coalesce via Frizzled5 in supporting cancer growth. Adv Sci. 2022;9:e2200750. 10.1002/advs.202200750 PMC9534957 35975457

[12] Zhu R, Ye X, Lu X, Xiao L, Yuan M, Zhao H, et al. ACSS2 acts as a lactyl-CoA synthetase and couples KAT2A to function as a lactyltransferase for histone lactylation and tumor immune evasion. Cell Metab. 2025;37:361–76.e7. 10.1016/j.cmet.2024.10.015 39561764

[13] Huang Y, Zhang R, Lyu H, Xiao S, Guo D, Chen XZ, et al. LncRNAs as nodes for the cross-talk between autophagy and Wnt signaling in pancreatic cancer drug resistance. Int J Biol Sci. 2024;20:2698–726. 10.7150/ijbs.91832 PMC11077374 38725864

[14] Li B, Xing F, Wang J, Wang X, Zhou C, Fan G, et al. YBX1 as a therapeutic target to suppress the LRP1-beta-catenin-RRM1 axis and overcome gemcitabine resistance in pancreatic cancer. Cancer Lett. 2024;602:217197. 10.1016/j.canlet.2024.217197 39216548

[15] Wei D, Yuan L, Xu X, Wu C, Huang Y, Zhang L, et al. Exploring epigenetic dynamics unveils a super-enhancer-mediated NDRG1-beta-catenin axis in modulating gemcitabine resistance in pancreatic cancer. Cancer Lett. 2024;605:217284. 10.1016/j.canlet.2024.217284 39366545

[16] Dewson G, Eichhorn PJA, Komander D. Deubiquitinases in cancer. Nat Rev Cancer. 2023;23:842–62. 10.1038/s41568-023-00633-y 37935888

[17] Dong S, Liang S, Cheng Z, Zhang X, Luo L, Li L, et al. ROS/PI3K/Akt and Wnt/beta-catenin signalings activate HIF-1alpha-induced metabolic reprogramming to impart 5-fluorouracil resistance in colorectal cancer. J Exp Clin Cancer Res. 2022;41:15. 10.1186/s13046-021-02229-6 PMC8742403 34998404

[18] Wang W, Shao F, Yang X, Wang J, Zhu R, Yang Y, et al. METTL3 promotes tumour development by decreasing APC expression mediated by APC mRNA N(6)-methyladenosine-dependent YTHDF binding. Nat Commun. 2021;12:3803. 10.1038/s41467-021-23501-5 PMC8217513 34155197

[19] Song P, Gao Z, Bao Y, Chen L, Huang Y, Liu Y, et al. Wnt/beta-catenin signaling pathway in carcinogenesis and cancer therapy. J Hematol Oncol. 2024;17:46. 10.1186/s13045-024-01563-4 PMC11184729 38886806

[20] Xiang Z, Wang Y, Ma X, Song S, He Y, Zhou J, et al. Targeting the NOTCH2/ADAM10/TCF7L2 axis-mediated transcriptional regulation of Wnt pathway suppresses tumor growth and enhances chemosensitivity in colorectal cancer. Adv Sci. 2025;12:e2405758. 10.1002/advs.202405758 PMC11744699 39601111

[21] Leung YK, Lee SG, Wang J, Guruvaiah P, Rusch NJ, Ho SM, et al. The loss of an orphan nuclear receptor NR2E3 augments Wnt/beta-catenin signaling via epigenetic dysregulation that enhances Sp1-beta catenin-p300 interactions in hepatocellular carcinoma. Adv Sci. 2024;11:e2308539. 10.1002/advs.202308539 PMC11304255 38790135

[22] Li B, Ming H, Qin S, Zhou L, Huang Z, Jin P, et al. HSPA8 activates Wnt/beta-catenin signaling to facilitate BRAF V600E colorectal cancer progression by CMA-mediated CAV1 degradation. Adv Sci. 2024;11:e2306535. 10.1002/advs.202306535 PMC10797426 37973552

[23] Harrigan JA, Jacq X, Martin NM, Jackson SP. Deubiquitylating enzymes and drug discovery: emerging opportunities. Nat Rev Drug Discov. 2018;17:57–78. 10.1038/nrd.2017.152 PMC7097658 28959952

[24] Butler LR, Densham RM, Jia J, Garvin AJ, Stone HR, Shah V, et al. The proteasomal de-ubiquitinating enzyme POH1 promotes the double-strand DNA break response. EMBO J. 2012;31:3918–34. 10.1038/emboj.2012.232 PMC3463844 22909820

[25] Yang P, Yang X, Wang D, Yang H, Li Z, Zhang C, et al. PSMD14 stabilizes estrogen signaling and facilitates breast cancer progression via deubiquitinating ERalpha. Oncogene. 2024;43:248–64. 10.1038/s41388-023-02905-1 PMC10798890 38017133

[26] Sun T, Liu Z, Bi F, Yang Q. Deubiquitinase PSMD14 promotes ovarian cancer progression by decreasing enzymatic activity of PKM2. Mol Oncol. 2021;15:3639–58. 10.1002/1878-0261.13076 PMC8637564 34382324

[27] Lv J, Zhang S, Wu H, Lu J, Lu Y, Wang F, et al. Deubiquitinase PSMD14 enhances hepatocellular carcinoma growth and metastasis by stabilizing GRB2. Cancer Lett. 2020;469:22–34. 10.1016/j.canlet.2019.10.025 31634528

[28] Jing T, Xu X, Wu C, Wei D, Yuan L, Huang Y, et al. POH1 facilitates pancreatic carcinogenesis through MYC-driven acinar-to-ductal metaplasia and is a potential therapeutic target. Cancer Lett. 2023;577:216444. 10.1016/j.canlet.2023.216444 37844756

[29] Seo D, Jung SM, Park JS, Lee J, Ha J, Kim M, et al. The deubiquitinating enzyme PSMD14 facilitates tumor growth and chemoresistance through stabilizing the ALK2 receptor in the initiation of BMP6 signaling pathway. EBioMedicine. 2019;49:55–71. 10.1016/j.ebiom.2019.10.039 PMC7113187 31685442

[30] Cooper EM, Cutcliffe C, Kristiansen TZ, Pandey A, Pickart CM, Cohen RE. K63-specific deubiquitination by two JAMM/MPN+ complexes: BRISC-associated Brcc36 and proteasomal Poh1. EMBO J. 2009;28:621–31. 10.1038/emboj.2009.27 PMC2666030 19214193

[31] Zhang L, Liu Y, Wang B, Xu G, Yang Z, Tang M, et al. POH1 deubiquitinates pro-interleukin-1beta and restricts inflammasome activity. Nat Commun. 2018;9:4225. 10.1038/s41467-018-06455-z PMC6185913 30315153

[32] Mani SKK, Yan B, Cui Z, Sun J, Utturkar S, Foca A, et al. Restoration of RNA helicase DDX5 suppresses hepatitis B virus (HBV) biosynthesis and Wnt signaling in HBV-related hepatocellular carcinoma. Theranostics. 2020;10:10957–72. 10.7150/thno.49629 PMC7532671 33042264

[33] Yin H, Gao T, Xie J, Huang Z, Zhang X, Yang F, et al. FUBP1 promotes colorectal cancer stemness and metastasis via DVL1-mediated activation of Wnt/beta-catenin signaling. Mol Oncol. 2021;15:3490–512. 10.1002/1878-0261.13064 PMC8637553 34288405

[34] Wald JH, Hatakeyama J, Printsev I, Cuevas A, Fry WHD, Saldana MJ, et al. Suppression of planar cell polarity signaling and migration in glioblastoma by Nrdp1-mediated Dvl polyubiquitination. Oncogene. 2017;36:5158–67. 10.1038/onc.2017.126 PMC5589482 28481871

[35] Maurice MM, Angers S. Mechanistic insights into Wnt-beta-catenin pathway activation and signal transduction. Nat Rev Mol Cell Biol. 2025;26:371–88. 10.1038/s41580-024-00823-y 39856369

[36] Shukla SK, Purohit V, Mehla K, Gunda V, Chaika NV, Vernucci E, et al. MUC1 and HIF-1alpha signaling crosstalk induces anabolic glucose metabolism to impart gemcitabine resistance to pancreatic cancer. Cancer Cell. 2017;32:392. 10.1016/j.ccell.2017.08.008 28898700

[37] Fendt SM. 100 years of the Warburg effect: a cancer metabolism endeavor. Cell. 2024;187:3824–8. 10.1016/j.cell.2024.06.026 39059359

[38] Rim EY, Clevers H, Nusse R. The Wnt pathway: from signaling mechanisms to synthetic modulators. Annu Rev Biochem. 2022;91:571–98. 10.1146/annurev-biochem-040320-103615 35303793

[39] Li J, Yakushi T, Parlati F, Mackinnon AL, Perez C, Ma Y, et al. Capzimin is a potent and specific inhibitor of proteasome isopeptidase Rpn11. Nat Chem Biol. 2017;13:486–93. 10.1038/nchembio.2326 PMC5570473 28244987

[40] Spataro V, Buetti-Dinh A. POH1/Rpn11/PSMD14: a journey from basic research in fission yeast to a prognostic marker and a druggable target in cancer cells. Br J Cancer. 2022;127:788–99. 10.1038/s41416-022-01829-z PMC9428165 35501388

