# Supplementary material for: Targeting PSMD14 increases chemotherapeutic sensitivity to gemcitabine by inhibiting the WNT/β-catenin pathway and glycolysis in pancreatic cancer
Source: Cell Death Dis. 2026 Aug 3;17(1):924. doi: 10.1038/s41419-026-08958-w (PMC13720168; doi:10.1038/s41419-026-08958-w)

Figure 1C

PSMD14

β-actin

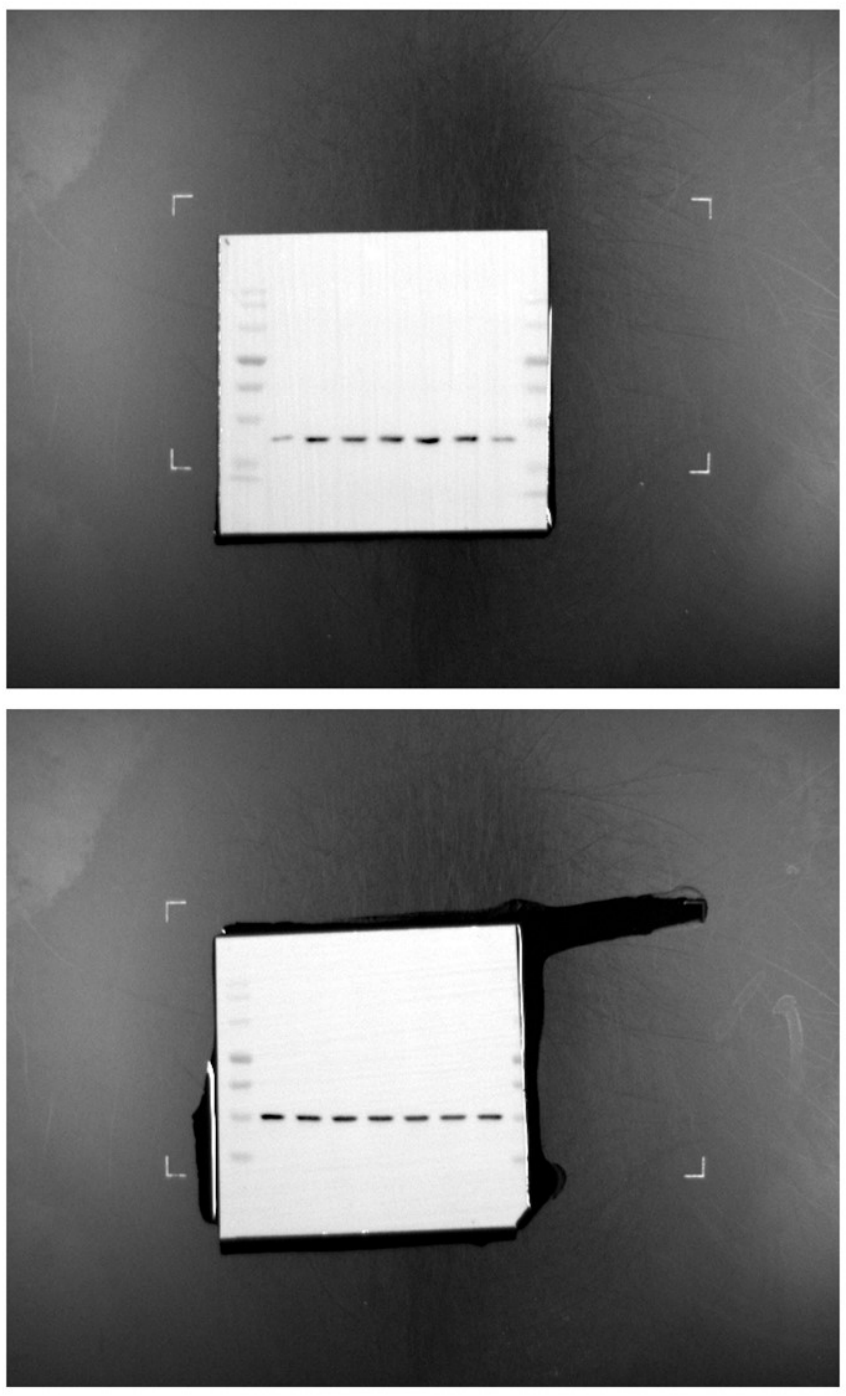

Figure 5D

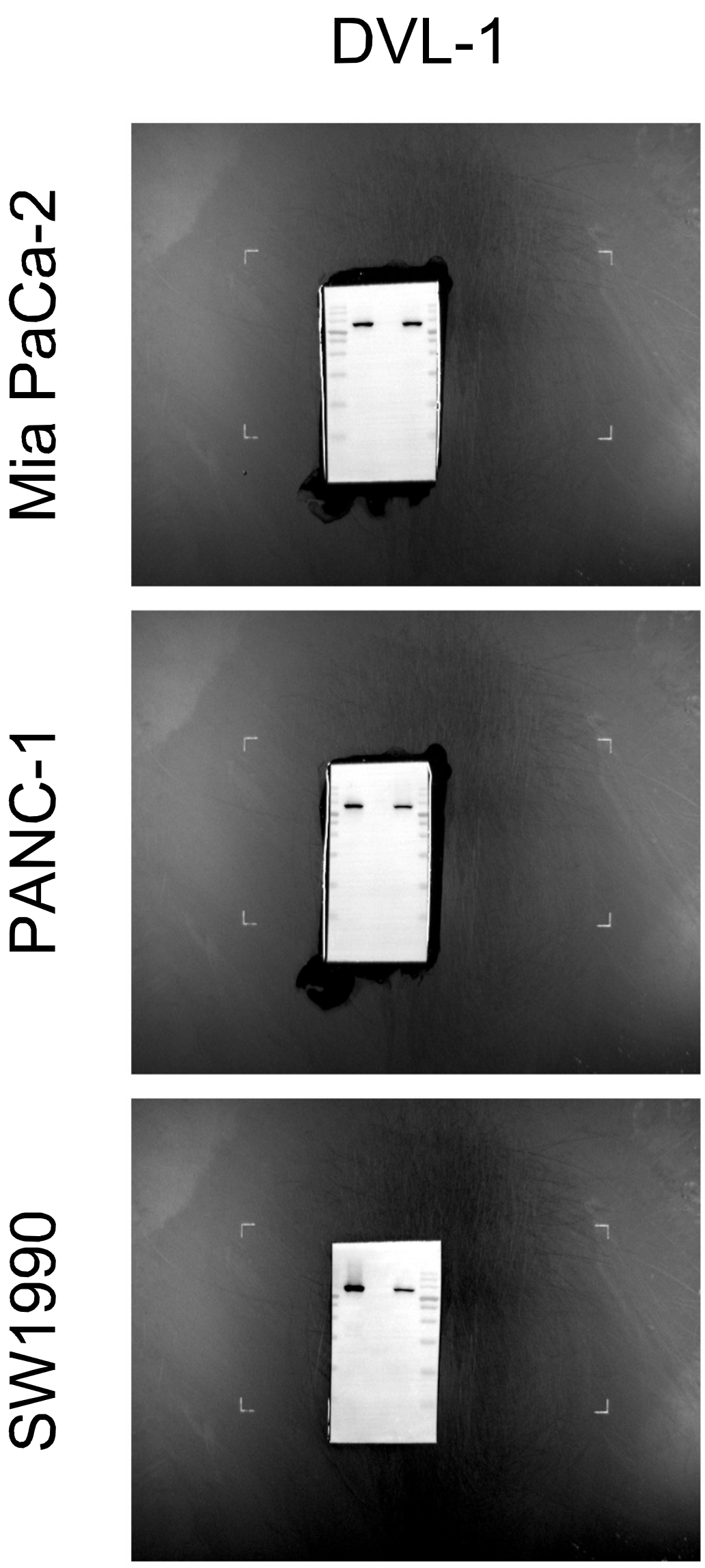

Figure 5G

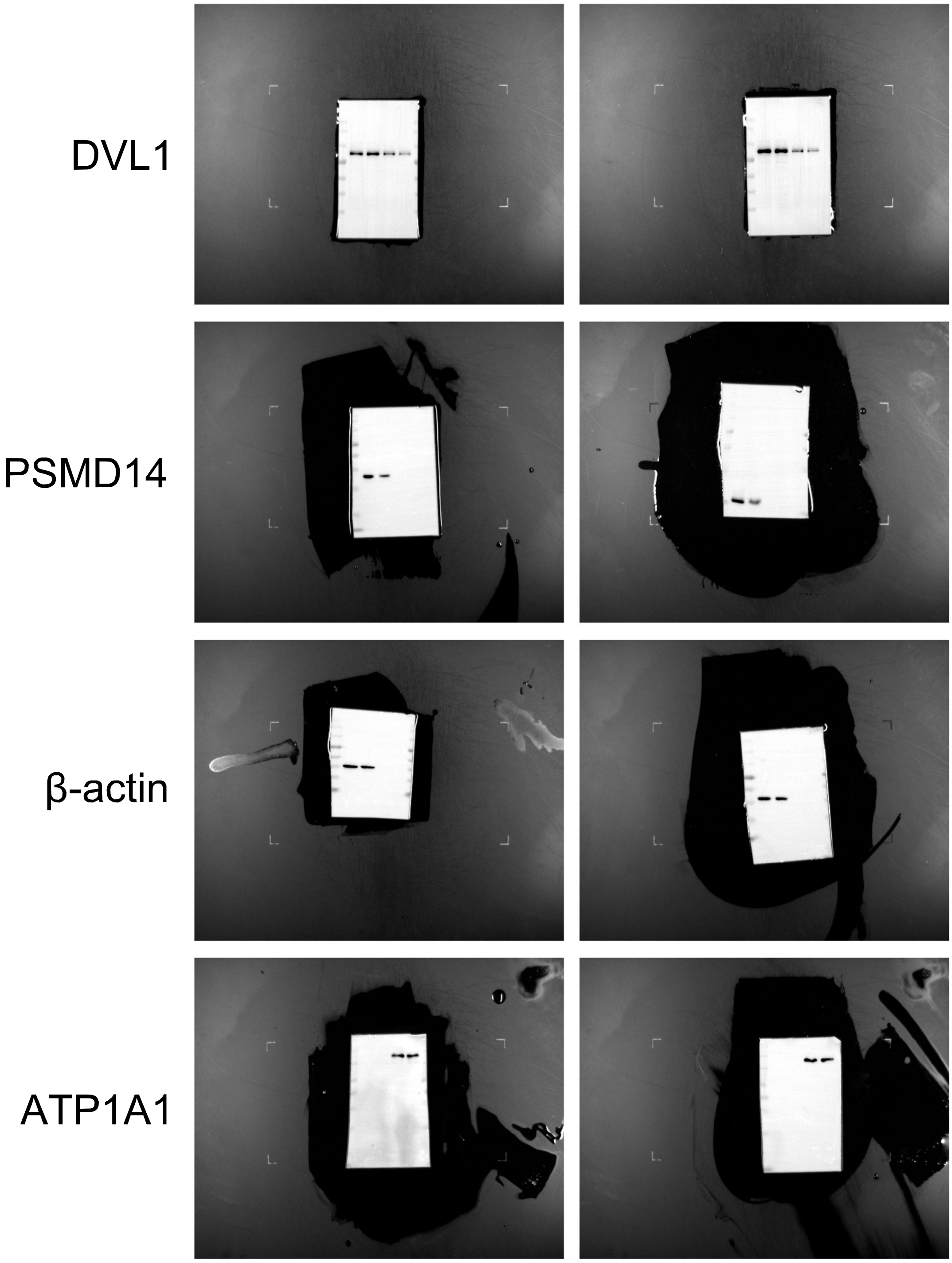

Figure 5I

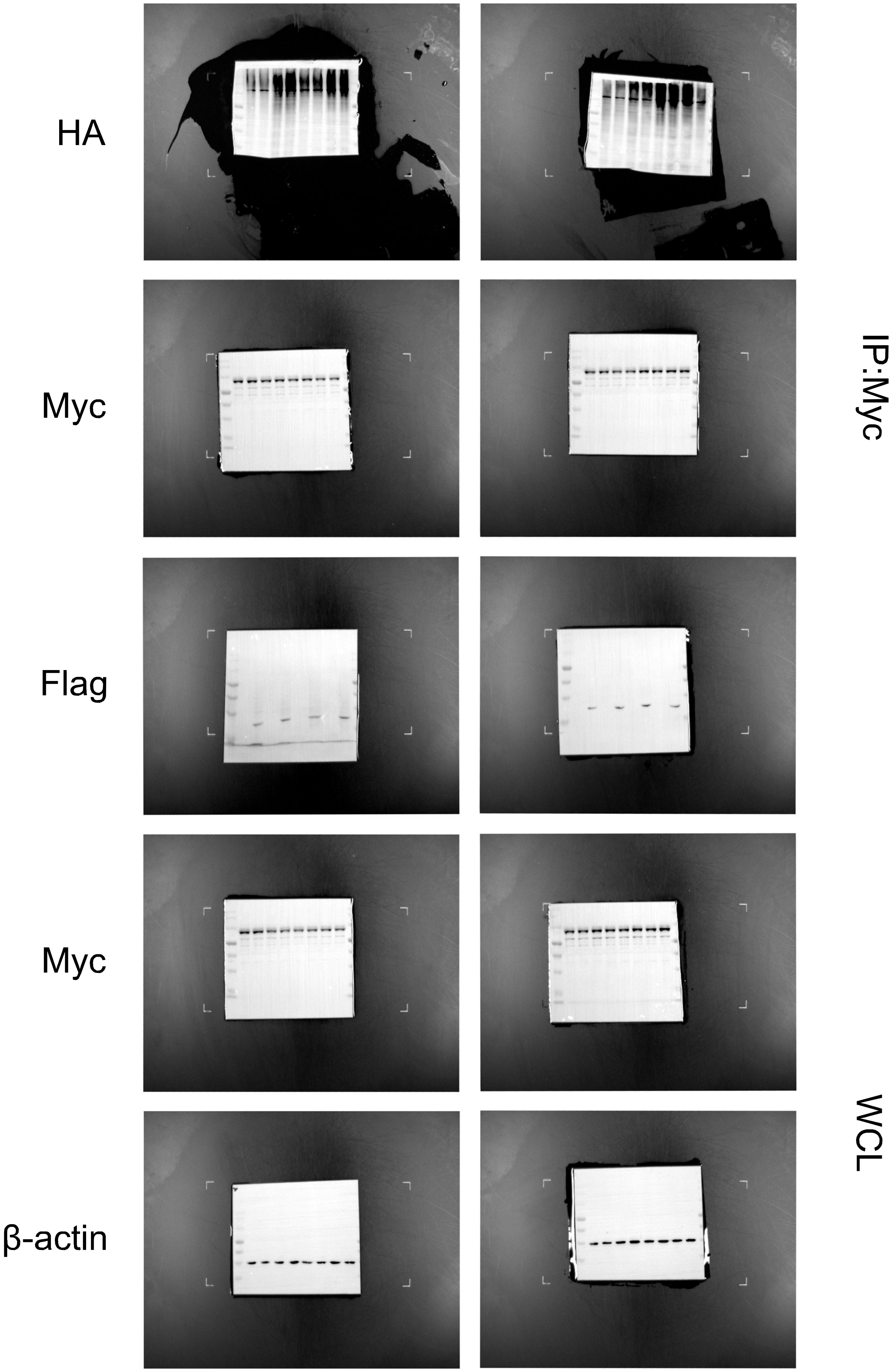

Figure 5J

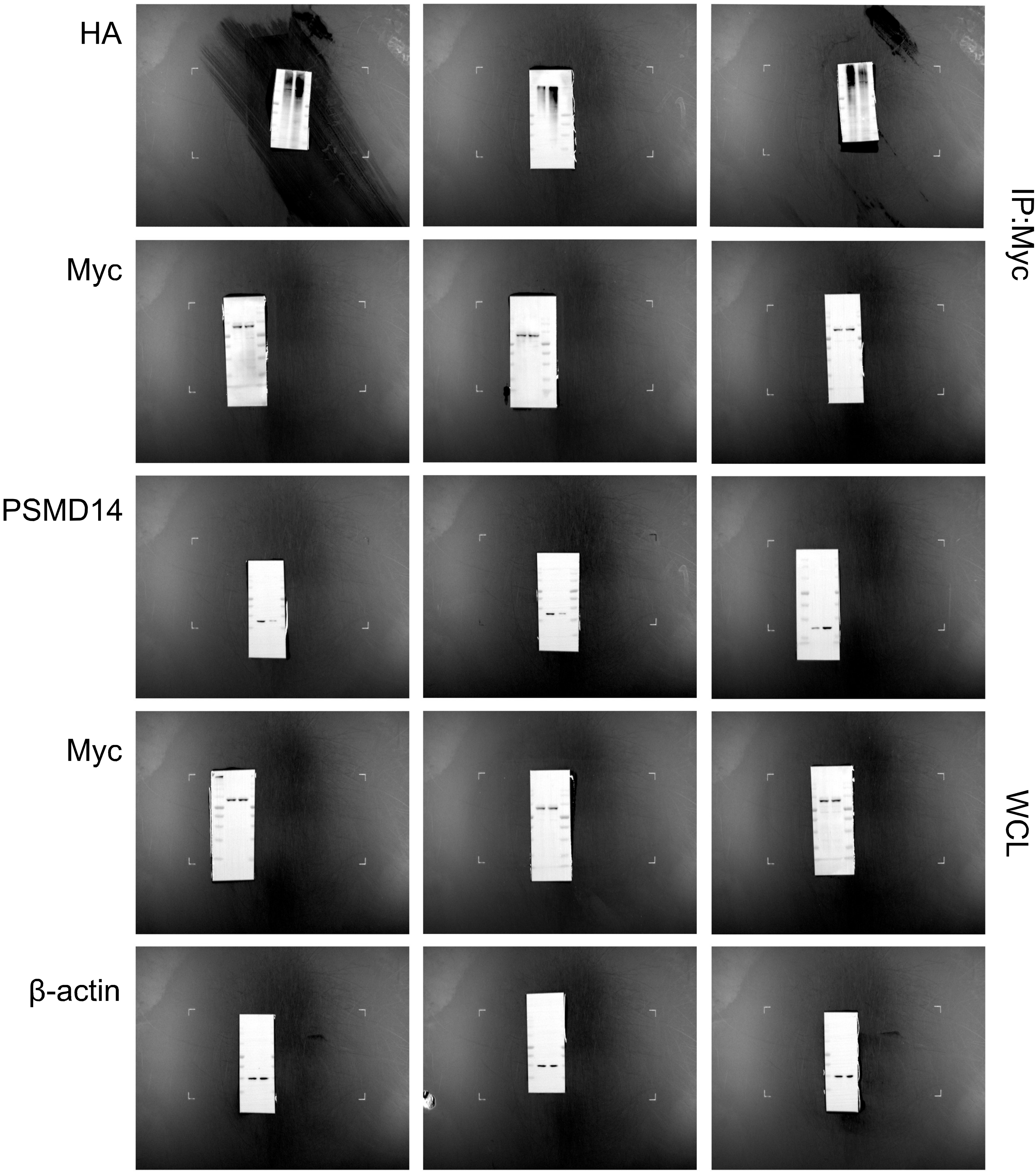

Figure 6A

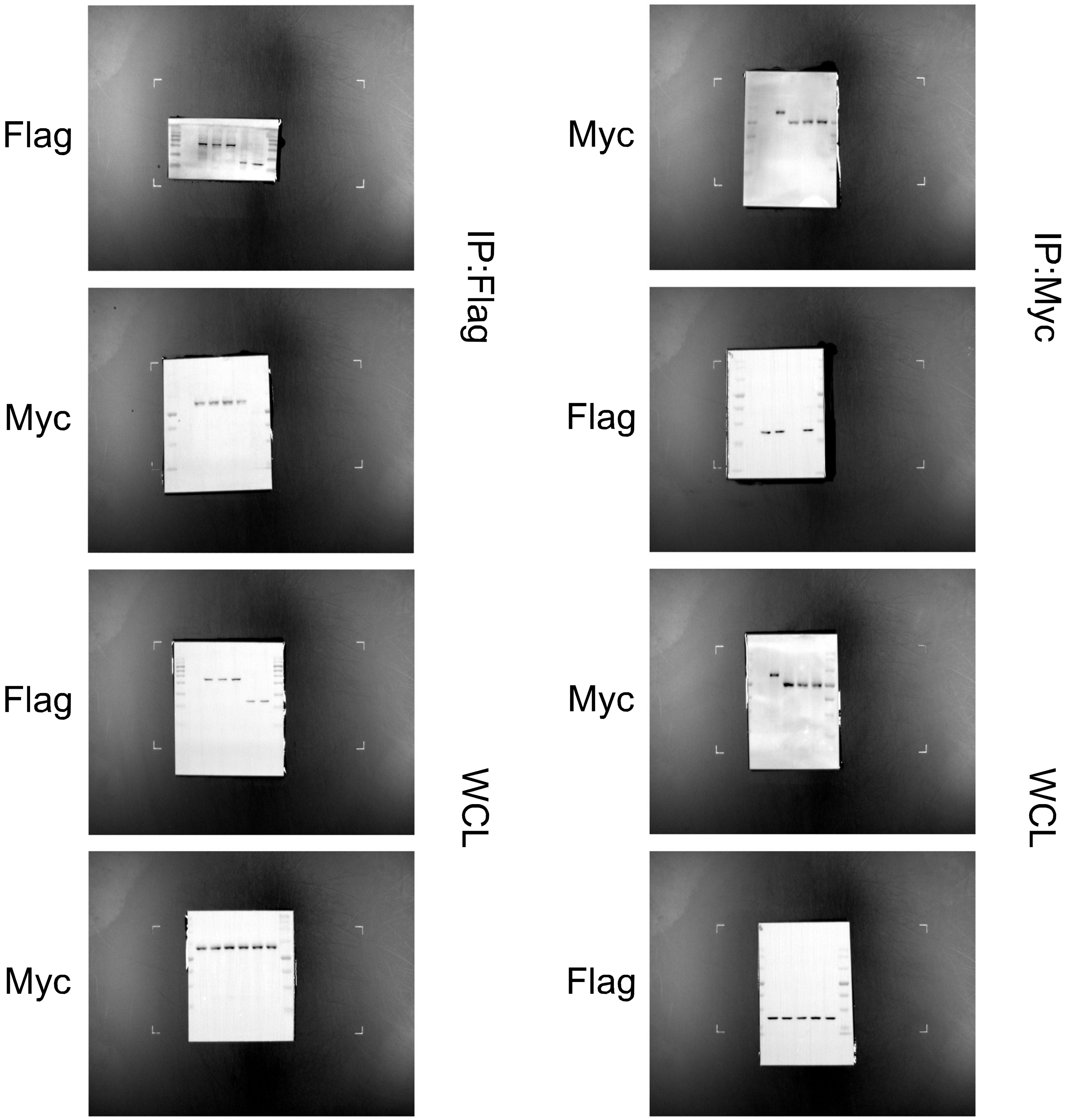

Figure 6B

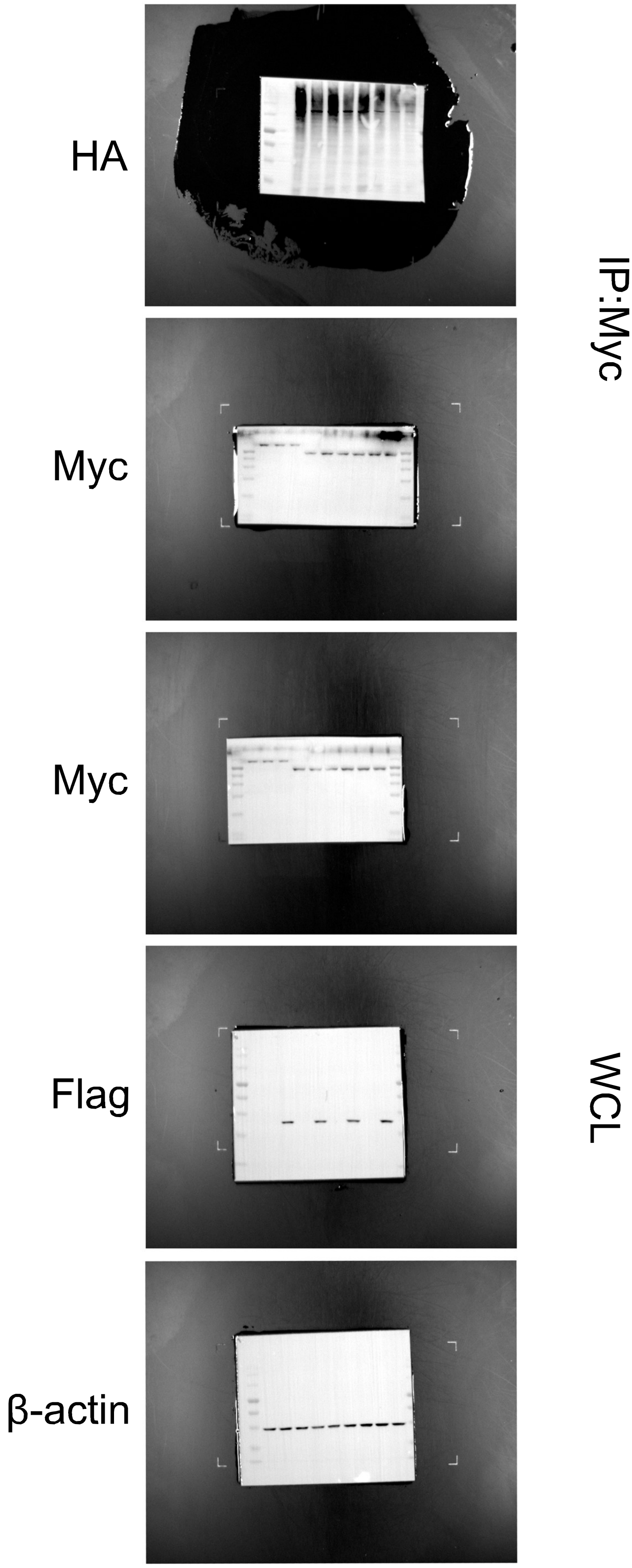

Figure 6C

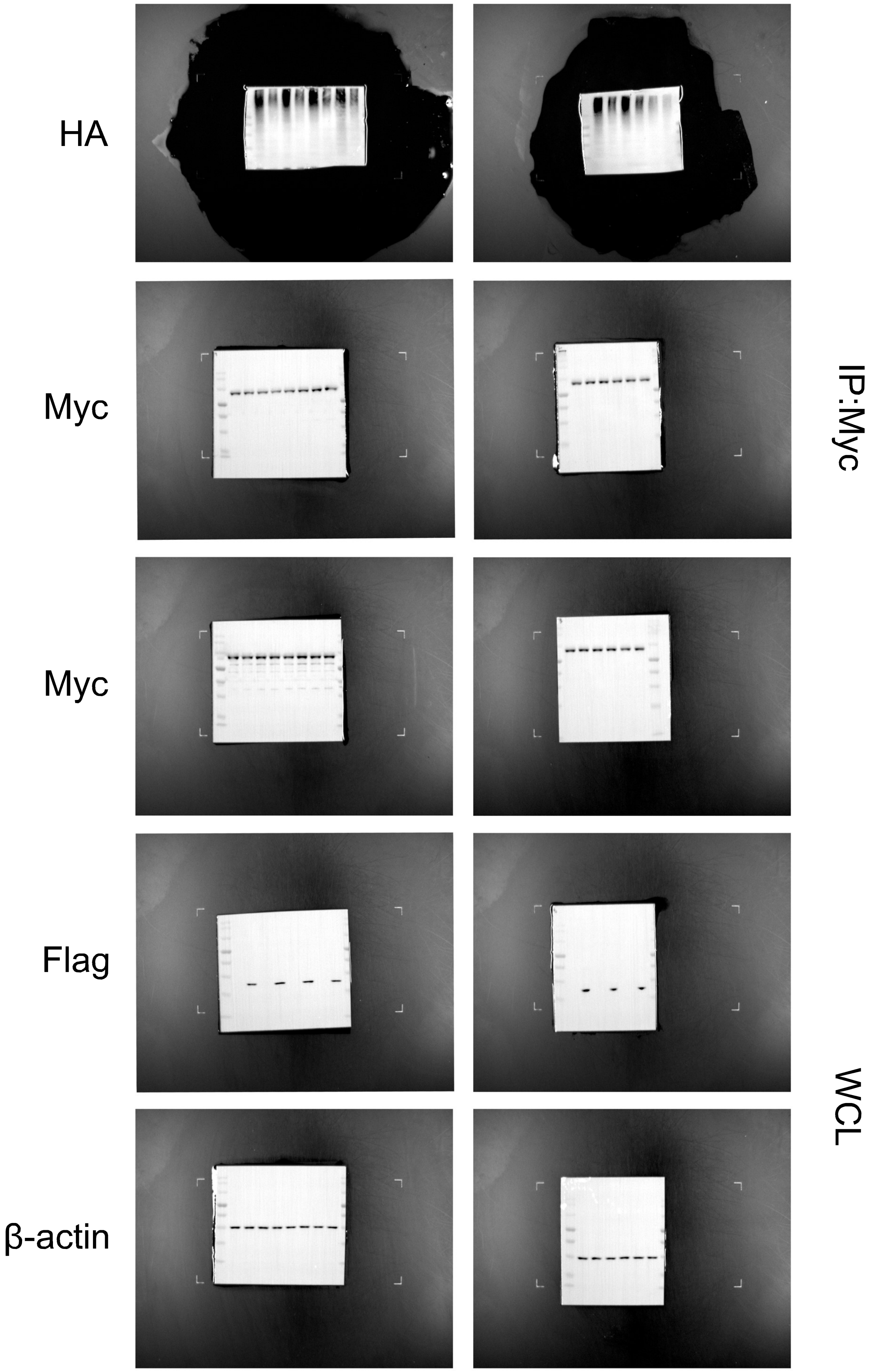

Figure 6D

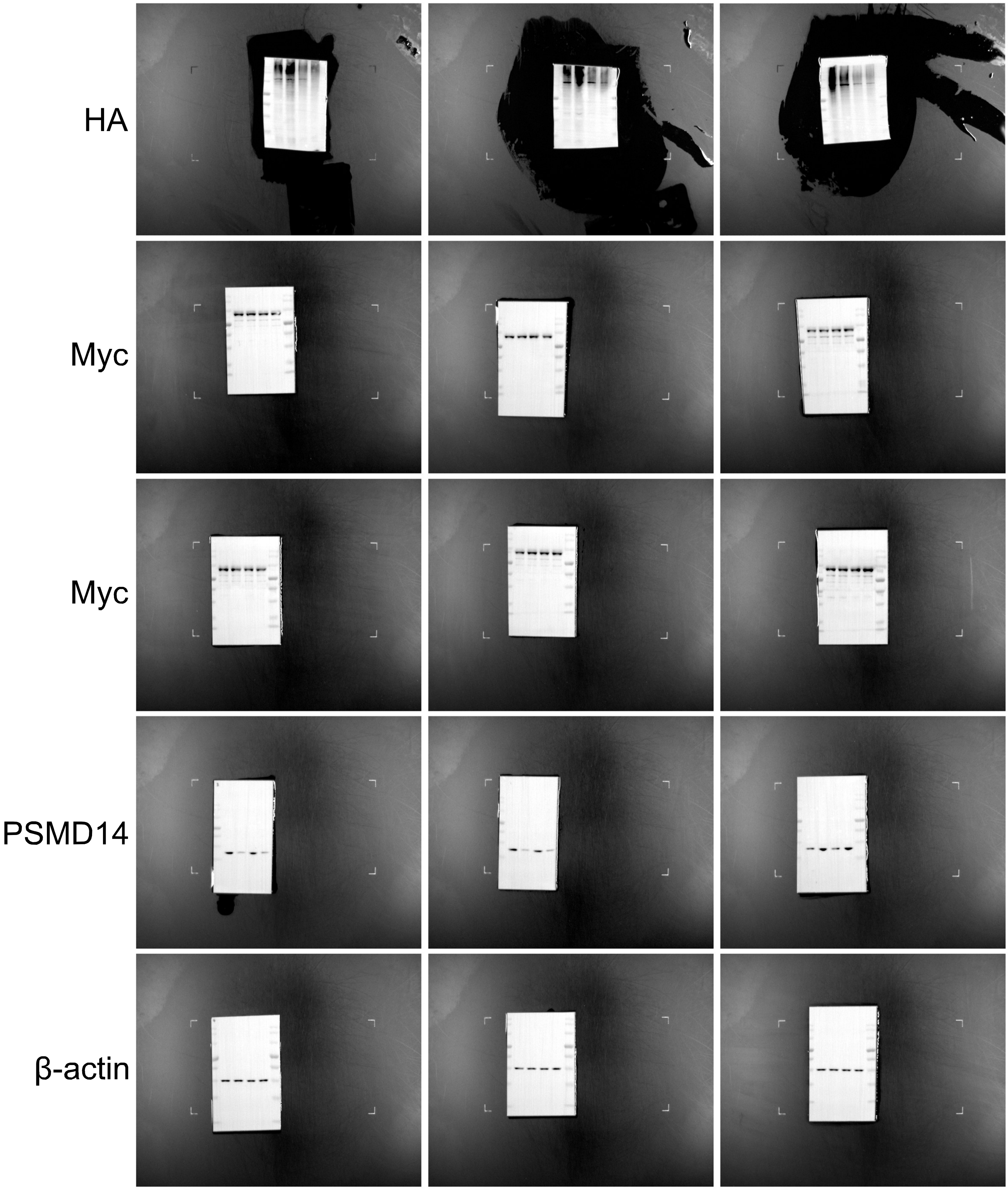

Figure 6E

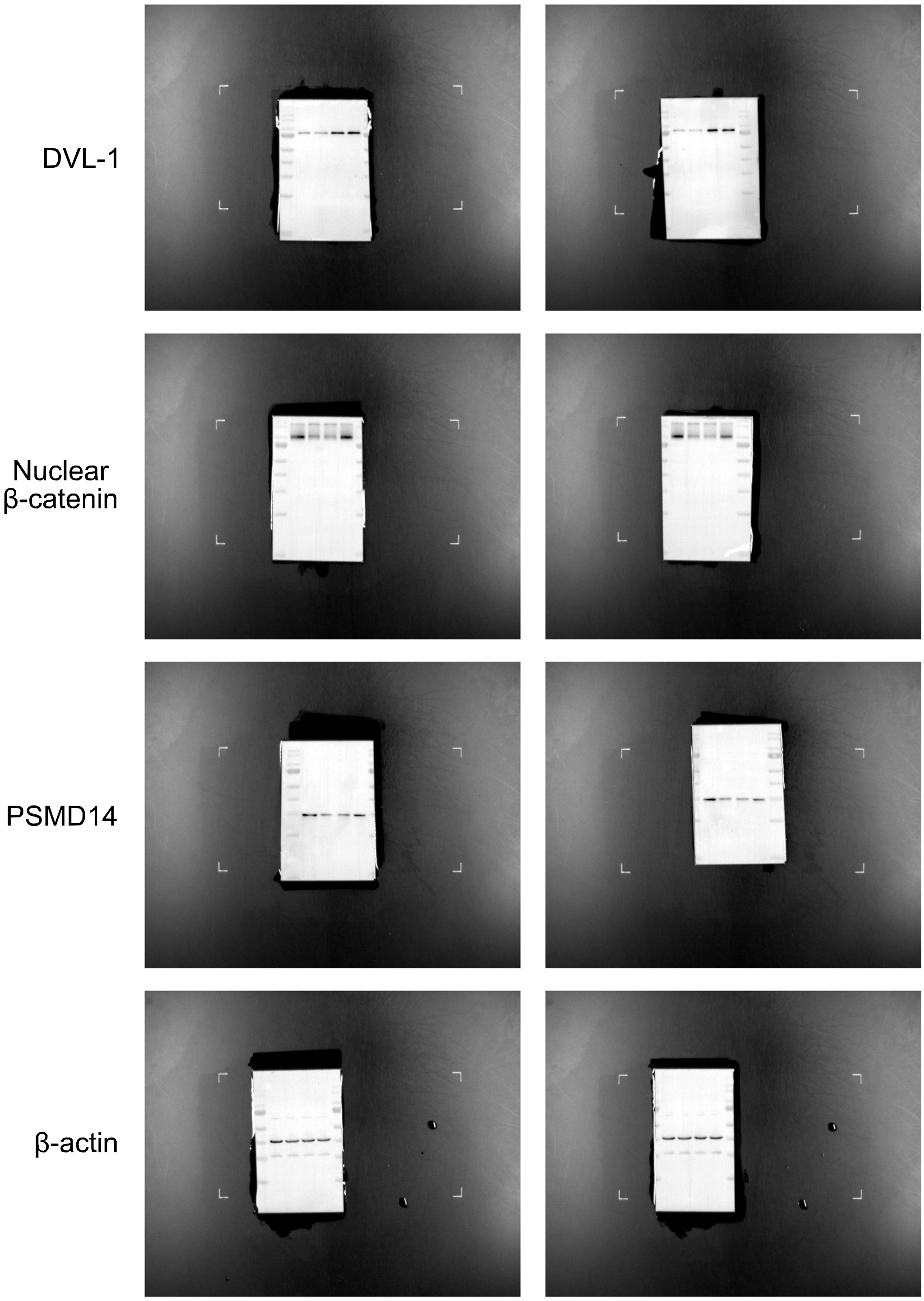

Figure 7H

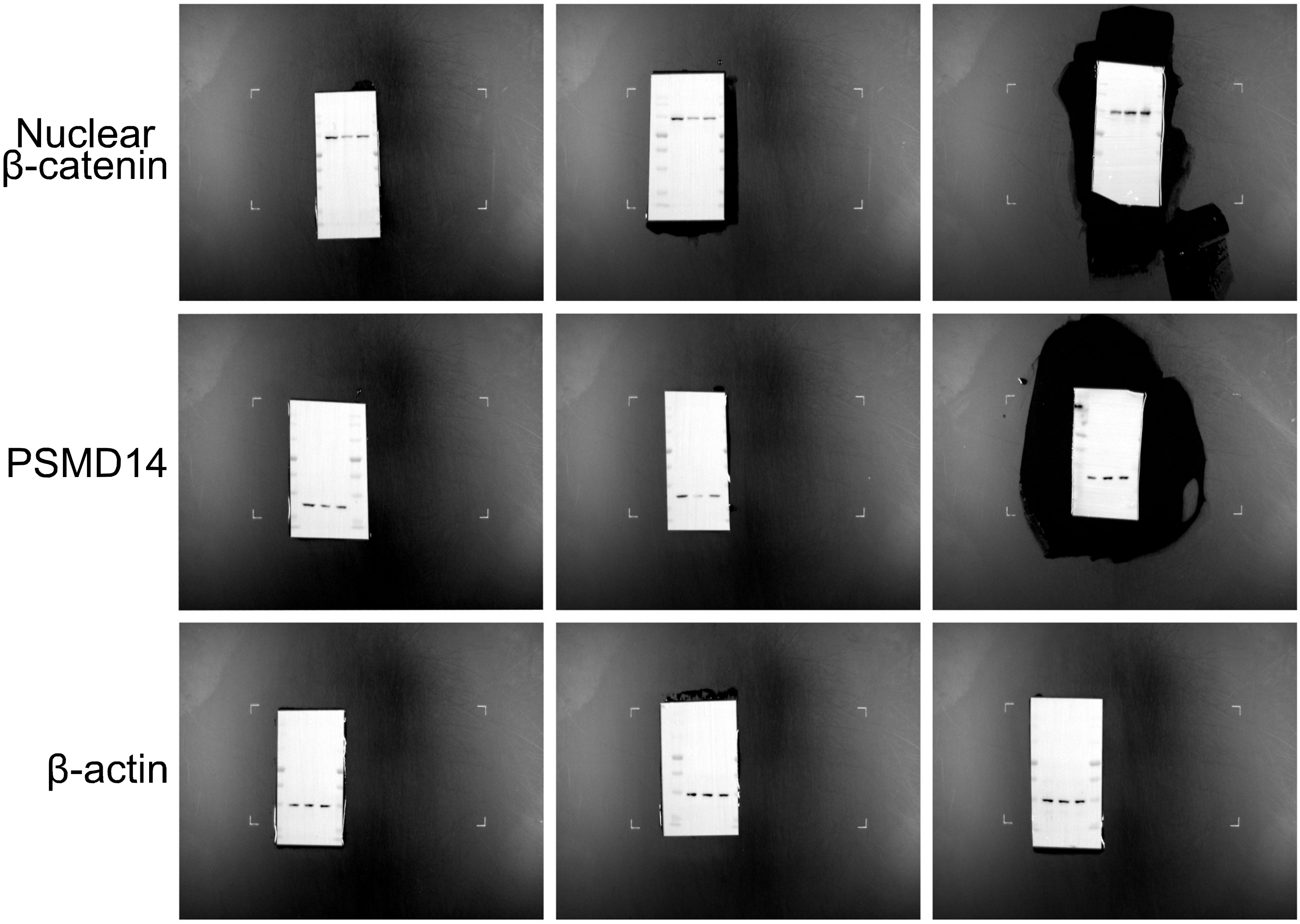

Figure S1

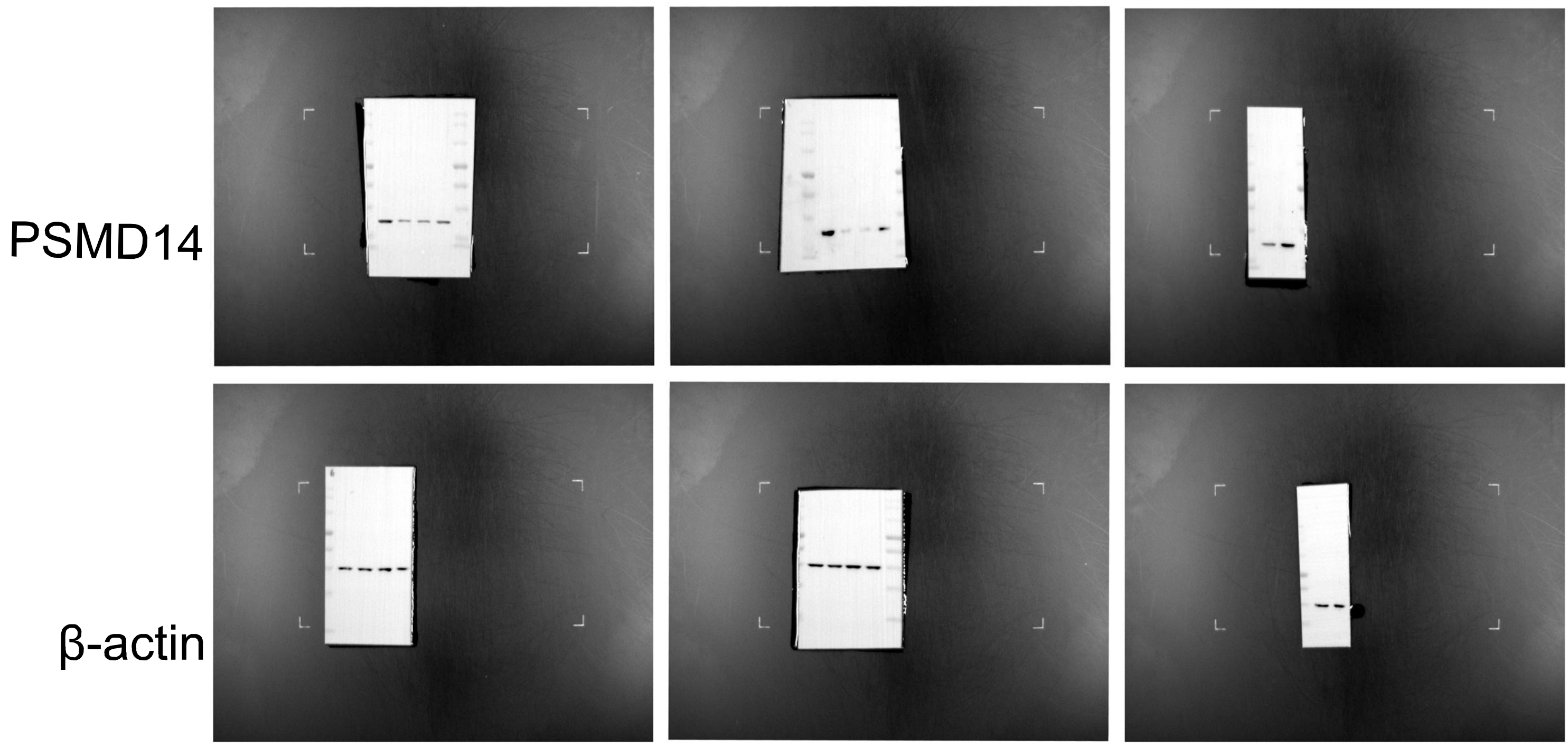

Figure S2D

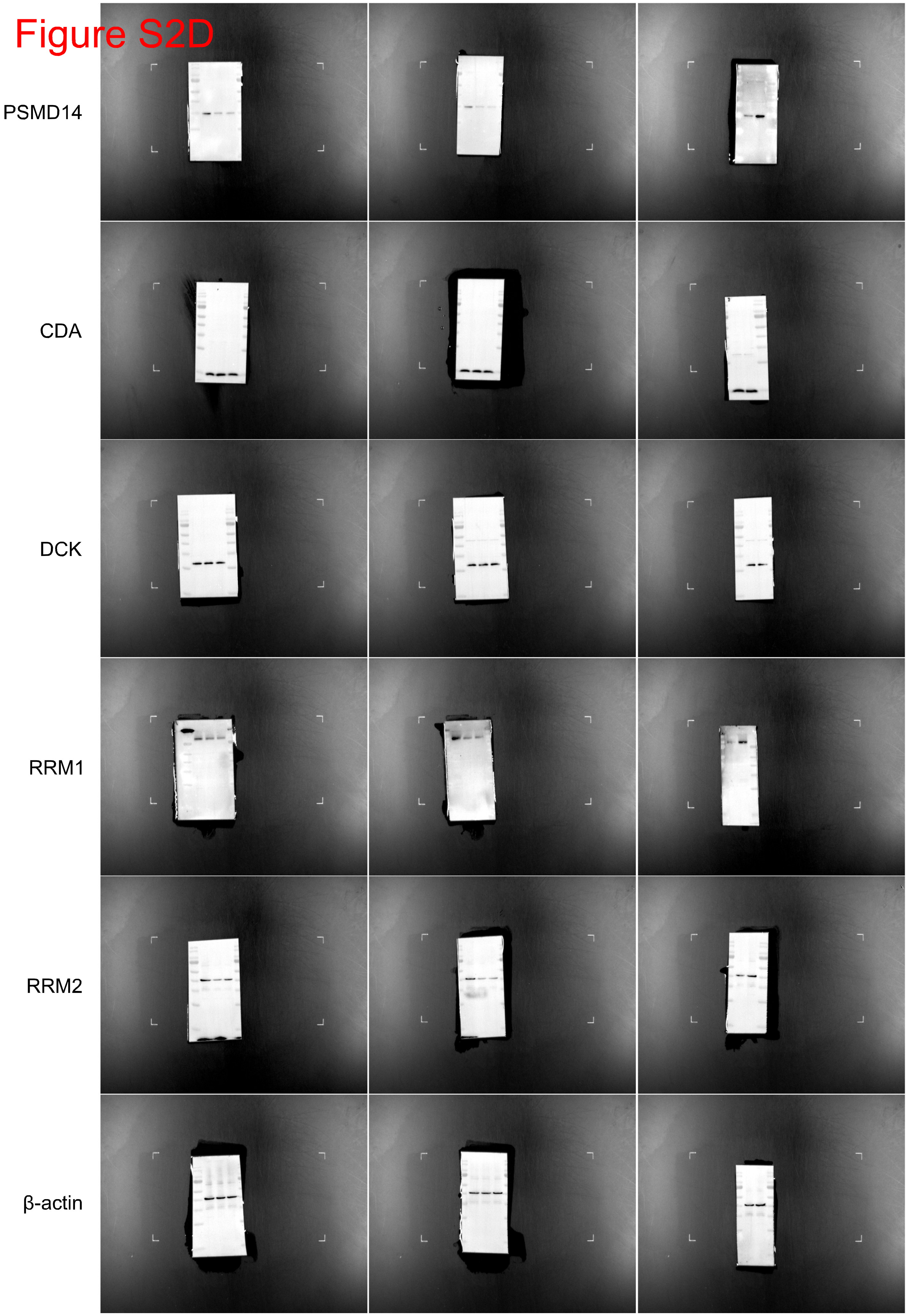

Figure S2H

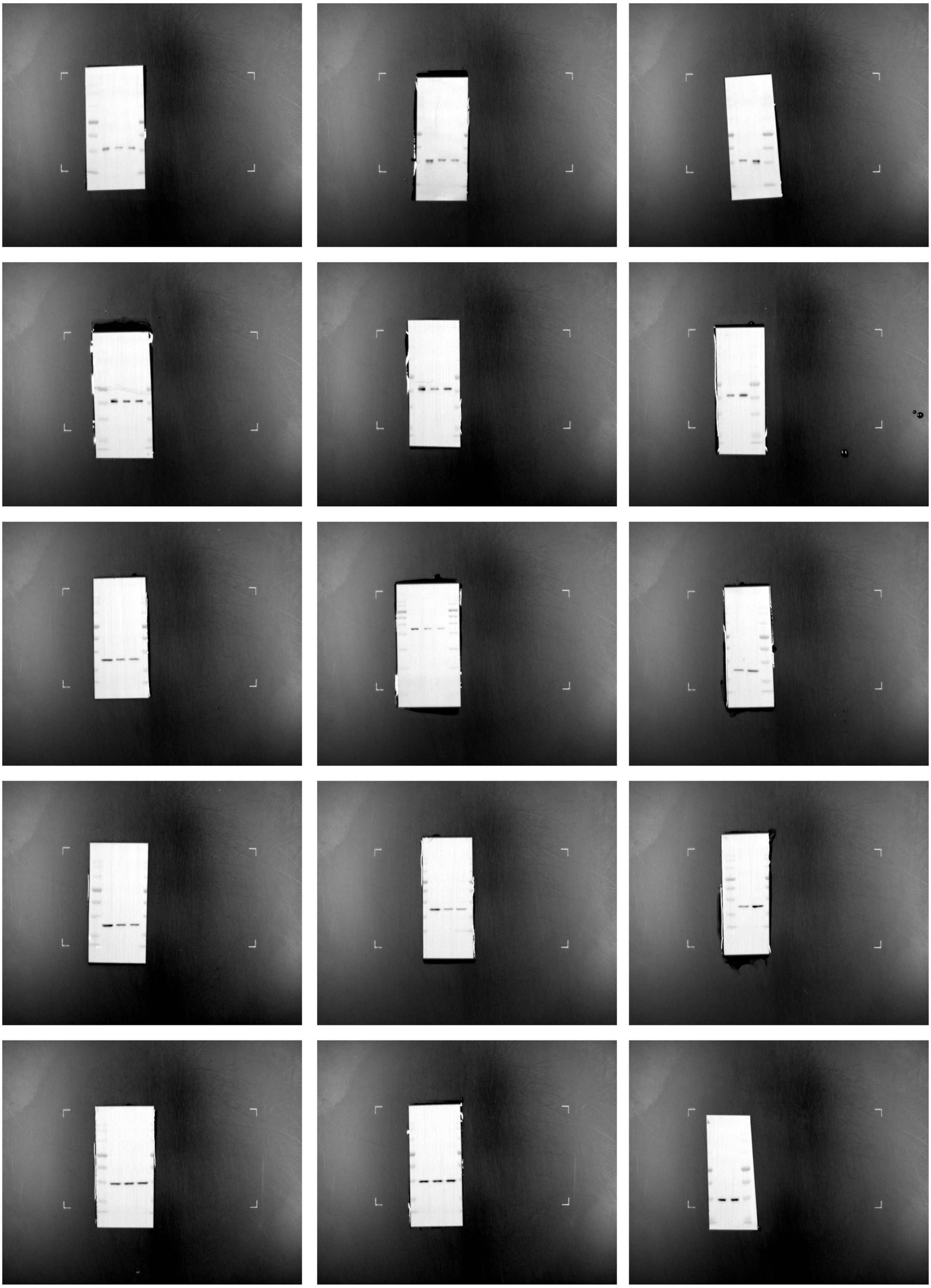

PDK1

PKM2

LDHA

PSMD14

β-actin

Figure S3D

PSMD14

Nuclear
β-catenin

RRM1

RRM2

β-actin

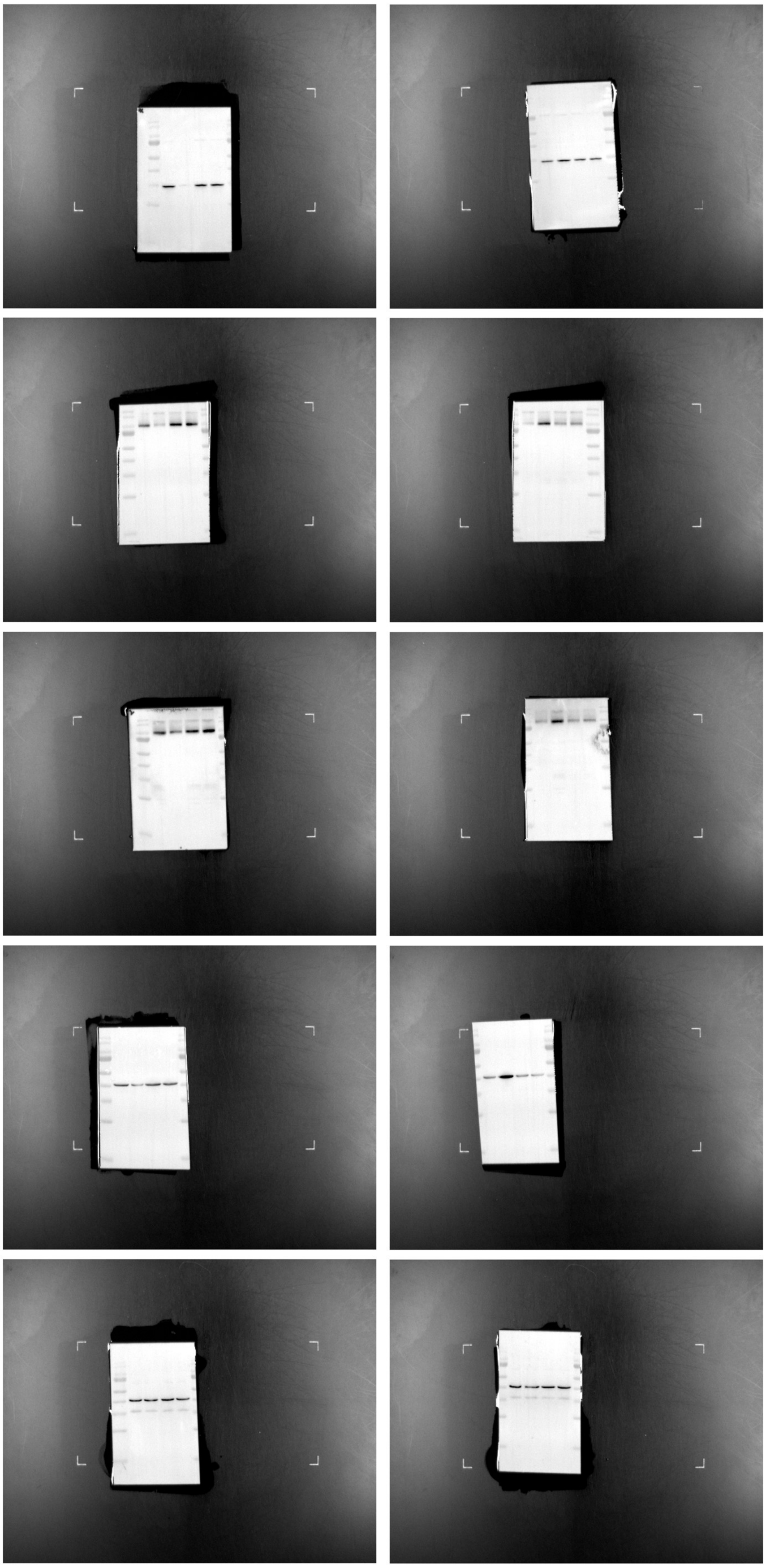

Figure S4A

PANC-1 Mia PaCa-2

DVL-1

β-actin

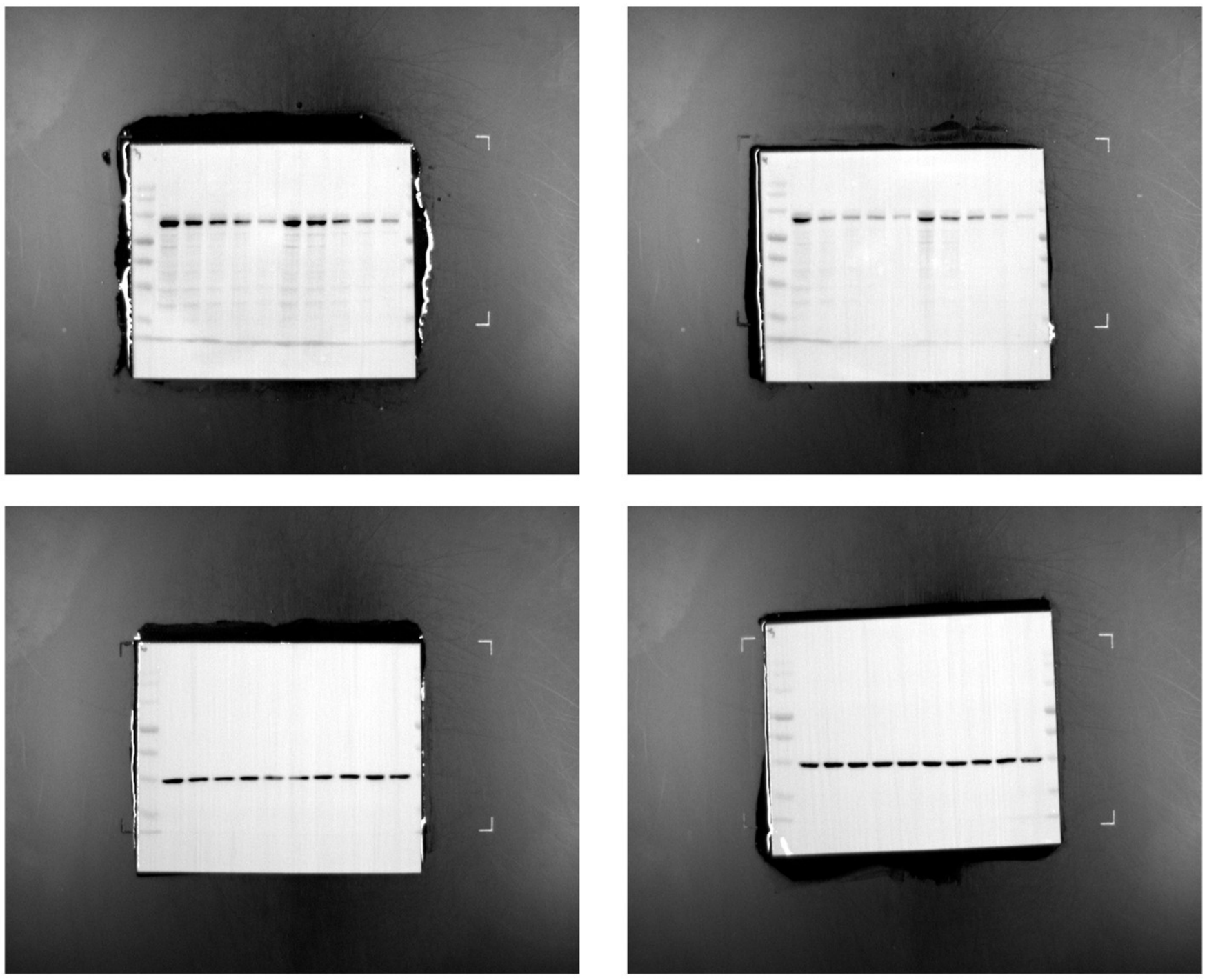

Figure S4B

RUVBL1

Mia PaCa-2

PANC-1

SW1990

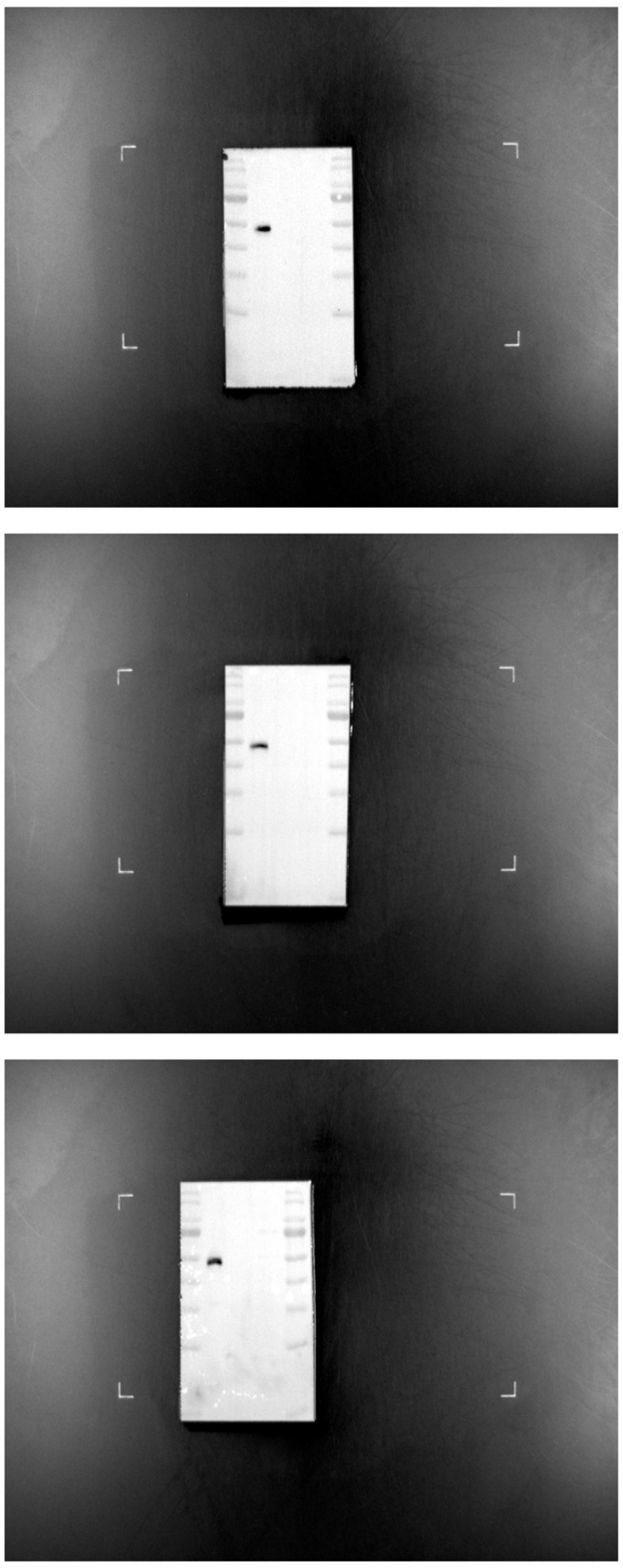

Supplement: Supplementary file 1 [file 41419_2026_8958_MOESM2_ESM.pdf]
