# Supplementary material for: Targeting PSMD14 increases chemotherapeutic sensitivity to gemcitabine by inhibiting the WNT/β-catenin pathway and glycolysis in pancreatic cancer
Source: Cell Death Dis. 2026 Aug 3;17(1):924. doi: 10.1038/s41419-026-08958-w (PMC13720168; doi:10.1038/s41419-026-08958-w)
Supplement: Supplementary file 2 [file 41419_2026_8958_MOESM1_ESM.docx]

**Supplementary figures and supplementary figure legends**

**Figure S1. Verification of lentivirus knock-in or knock-down cell lines**

(A) The knock-in or knock-down and control cell lines were verified using RT-qPCR (n=3). (B) The knock-in or knock-down and control cell lines were verified using Western blot. * *p* < 0.05, ** *p* < 0.01.

**Figure S2. PSMD14 promotes PC glycolysis and induces insensitivity to gemcitabine**

(A) Glucose uptake assay was conducted to evaluate cells’ glucose uptake ability (n=3). (B) L-lactate production assay was conducted to evaluate cells’ lactate production ability (n=3). (C) IF was conducted to assess the γ-H2AX expression in cells of different group (n=3). (D) The expression of several enzymes involved in gemcitabine metabolism was detected by Western blot. (E) The expression levels of PSMD14 in PC cells and drug-resistant PC cells were analyzed based on the GSE140077 and GSE148200 datasets (n=3). (F) The expression levels of glycolysis related enzymes in PC cells were analyzed using RT-qPCR (n=3). (G) The correlations between PSMD14 and PDK1, PKM and LDHA transcriptional levels in the TCGA and GTEx databases were analyzed using the online website GEPIA. (H) The expression of PDK1, PKM and LDHA in PC cells were analyzed using Western blot. (I) The sensitivity experiment of gemcitabine was adopted when PC cells were treated with 2DG or interfered with LDHA expression (n=5). * *p* < 0.05, ** *p* < 0.01.

**Figure S3.** **Inhibition of the Wnt/β-catenin signaling pathway restricts the function of PSMD14**

(A) Glucose uptake assay and L-lactate production assay were conducted to evaluate the glycolysis of PANC-1 cells (n=3). (B) Glucose uptake assay and L-lactate production assay were conducted to evaluate the glycolysis of SW1990 cells (n=3). (C) Statistical result of Calcein-AM/PI (n=6). (D) The expression of nuclear catenin, RRM1, and RRM2 in each group of cells was detected using Western blot. (E) IF was conducted to assess the γ-H2AX expression in cells of different groups (n=3). (F) The expression of PSMD14 in various organoids was detected using IHC. * *p* < 0.05, ** *p* < 0.01.

**Figure S4.** **PSMD14 removes the K63-linked ubiquitin chain of DVL1 and facilitates its membrane trafficking**

(A) The immunoprecipitation experiment was used to verify whether there is an interaction between PSMD14 and RUVBL1. (B) The stability of DVL1 protein was detected by Western blot.

**Figure S5.** **Activation of the WNT/β-catenin pathway promotes PSMD14 transcription**

(A) The correlations between PSMD14 and TCF7L1, TCF7L2 and LEF1 transcriptional levels in the TCGA and GTEx databases were analyzed using the online website GEPIA.

**Figure S6.** **Targeting PSMD14 sensitizes PC to gemcitabine**

(A) The mortality rate of PC cells was detected using Calcein AM/PI, scare bar = 200μm. (B) Proteasome activity assay was used to evaluate the protease activity of PC cells (n=3). (C) ECAR assay was conducted to evaluate the glycolysis of PC cells (n=3). (D) Glucose uptake assay was conducted to evaluate the glycolysis of PC cells (n=3). (E) L-lactate production assay was conducted to evaluate the glycolysis of PC cells (n=3). (F) TOP/FOP Flash assay was conducted to evaluate the transcriptional activity of WNT/β-catenin pathway (n=3). * *p* < 0.05, ** *p* < 0.01.

**Supplementary tables**

**Table S1** Antibodies list.

| Antibody | Manufacturer | Cat No. |
| --- | --- | --- |
| PSMD14 | Proteintech | #12059-1-AP |
| β-actin | Proteintech | #66009-1-Ig |
| β-catenin | Proteintech | #51067-2-AP |
| LDHA | Proteintech | #19987-1-AP |
| PDK1 | Proteintech | #18262-1-AP |
| PKM2 | Proteintech | #15822-1-AP |
| DVL1 | Sigma | #D3570 |
|  | Santa Cruz | #sc-8025 |
| RUVBL1 | Proteintech | #10210-2-AP |
|  | Abcam | #ab51500 |
| Flag-tag | Proteintech | #20543-1-AP |
| HA-tag | Proteintech | #51064-2-AP |
| Myc-tag | Proteintech | #60003-2-Ig |
| ATP1A1 | Proteintech | #14418-1-AP |
| ubiquitin | Proteintech | #10201-2-AP |
| TCF7L1 | Invitrogen | #PA5-40327 |
| TCF7L2 | Cell Signaling Technology | #2569 |
| LEF1 | Cell Signaling Technology | #76010 |
| KI67 | Proteintech | #27309-1-AP |

**Table S2.** Target sequences list.

| sh-RNA or si-RNA | Target sequence（5' to 3'） |
| --- | --- |
| sh-PSMD14#1 | CATGGACTAAACAGACATTAT |
| sh-PSMD14#2 | CAGTGAACATTGTAAACACAA |
| sh-PSMD14#3 | GTTGGCTTTCTGGTGTGGATA |
| sh-*Psmd14* | CGGACTAAACAGACATTATTA |
| si-CTNNB1 | AGGTGCTATCTGTCTGCTCTA |

**Table S3.** Primers list.

| Gene or Region | Primer | Sequence (5' to 3') |
| --- | --- | --- |
| PSMD14 | Forward primer | AAGTTATGGGTTTGATGCTTGGA |
|  | Reverse primer | ATACCAACCAACAACCATCTCC |
| β-actin | Forward primer | CATGTACGTTGCTATCCAGGC |
|  | Reverse primer | CTCCTTAATGTCACGCACGAT |
| GLUT1 | Forward primer | GGCCAAGAGTGTGCTAAAGAA |
|  | Reverse primer | ACAGCGTTGATGCCAGACAG |
| HK2 | Forward primer | GAGCCACCACTCACCCTACT |
|  | Reverse primer | CCAGGCATTCGGCAATGTG |
| PKM | Forward primer | ATGTCGAAGCCCCATAGTGAA |
|  | Reverse primer | TGGGTGGTGAATCAATGTCCA |
| ENO1 | Forward primer | AAAGCTGGTGCCGTTGAGAA |
|  | Reverse primer | GGTTGTGGTAAACCTCTGCTC |
| PDK1 | Forward primer | CTGTGATACGGATCAGAAACCG |
|  | Reverse primer | TCCACCAAACAATAAAGAGTGCT |
| LDHA | Forward primer | ATGGCAACTCTAAAGGATCAGC |
|  | Reverse primer | CCAACCCCAACAACTGTAATCT |
| Region 1 | Forward primer | AAGGAATCAGACCGAAGGCG |
|  | Reverse primer | TGGGCTCACTGCAACATC |
| Region 2 | Forward primer | TCCTGGACATCCGCTTGA |
|  | Reverse primer | CCGCGATATGGACCCTTG |
